# Supplementary material for: The level of adherence to best-practice guidelines by interprofessional teams with and without acute care nurse practitioners in cardiac surgery: A study protocol
Source: PLoS One. 2023 Mar 1;18(3):e0282467. doi: 10.1371/journal.pone.0282467 (PMC9976998; doi:10.1371/journal.pone.0282467)
Supplement: S2 Appendix — (DOCX) [file pone.0282467.s002.docx]

**SB Appendix**

**Diagnosis codes from the Canadian Classification of Health Information**

| **Type of surgery** | **Intervention codes** |
| --- | --- |
| Coronary artery bypass graft | 1.IJ.76.^^: Bypass, coronary arteries |
| Valve or annulus procedures | 1.HS.80.^^: Repair, tricuspid valve 1.HS.90.^^: Total excision with reconstruction, tricuspid valve  1.HT.80.^^: Repair, pulmonary valve  1.HT.90.^^: Total excision with reconstruction, pulmonary valve  1.HU.80.^^: Repair, mitral valve  1.HU.90.^^: Total excision with reconstruction, mitral valve  1.HV .80.^^: Repair, aortic valve  1.HV .90.^^: Total excision with reconstruction, aortic valve  1.HW.^^.^^: Therapeutic interventions on the annulus |
| (CIHI, 2019) | |
