# Supplementary material for: The level of adherence to best-practice guidelines by interprofessional teams with and without acute care nurse practitioners in cardiac surgery: A study protocol
Source: PLoS One. 2023 Mar 1;18(3):e0282467. doi: 10.1371/journal.pone.0282467 (PMC9976998; doi:10.1371/journal.pone.0282467)
Supplement: S3 Appendix — (DOCX) [file pone.0282467.s003.docx]

**SC Appendix**

**Variables under study**

| **Variable** | **Definition** | **Modality** | **Measure** |
| --- | --- | --- | --- |
| **Independent variable: interprofessional teams** | | | |
| Interprofessional teams within acute care postoperative cardiac surgery setting | A team composed of several health care professionals (HCP) in charge of the clinical and psychosocial condition of patients | Interprofessional teams with and without the inclusion of an acute care nurse practitioner (ACNP)  Interprofessional teams with ACNP: degree of implication of the ACNP in the care given to patients during their hospitalization at the postoperative cardiac surgery unit | Dichotomous variable:  0=team without an ACNP  1=team with an ACNP  Descriptive data:  The proportion of days of hospitalization of the patient under the care of the ACNP of the entire hospitalization at the postoperative cardiac surgery unit |
| **Dependant variable: level of adherence to best-practice guidelines by interprofessional teams** | | | |
| **Prescription and monitoring of the pharmacotherapy during hospitalization and at discharge** | | | |
| Anticoagulants  Beta Blockers  Lipid-lowering agents  Anti-Platelet agents | Prescription of at least one anticoagulant, beta blocker, lipid-lowering agent, and anti-platelet agent during the patient’s hospitalization at the postoperative cardiac surgery unit and upon hospital discharge, unless contraindicated | Minimum of one prescription of the medication during the patient hospitalization, and upon hospital discharge  Interventions performed by HCP associated to these prescriptions | Adherence to best-practice guidelines: score on 2 ( /2)  Dichotomous variable:  0= absence of at least one medication prescribed during hospitalization and at hospital discharge  1= prescription of at least one or more medication during hospitalization and at the hospital discharge  Dichotomous variable:  0= no interventions performed by HCP associated to these prescriptions  1= interventions performed by HCP associated with these prescriptions  Descriptive data:  Type of medication prescribe  The presence of contraindication |
| **Prescription and control of laboratory tests** | | | |
| Potassium (K+)  Magnesium (Mg+)  Serum glucose  International Normalized Ratio (INR) | The value of the laboratory test is within the normal range supported by the existing literature  Normal range:  K+: value equal or above 4mg/dl  Mg+:value equal or above 2mg/dl  Serum glucose: value equal or below 180mg/dl  INR: value between 2-3 (atrial fibrillation) and/or 2.5-3.5 (patient who underwent a valve repair) | Proportions of laboratory test values within the normal range  Proportion of abnormal laboratory test values  Associated interventions performed by HCP | Adherence to best-practice guidelines: score on 2 ( /2)  Dichotomous variable:  0=presence of abnormal values within the laboratory tests  0=absence of abnormal values within the laboratory tests  Dichotomous variable:  0=absence of interventions performed by HCP associated with the laboratory tests  1=presence of interventions performed by HCP associated with the laboratory tests  Descriptive data:  The number of prescriptions ordered by HCP during the patient hospitalization at the postoperative cardiac surgery unit  The average value of the laboratory test performed |
| **Postoperative assessment** | | | |
| Pain  Surgical wounds  Nutrition  Mobilization | Postoperative assessment of the patient’s pain, surgical wounds, nutrition, and mobilization at least once per day during the hospitalization at the postoperative cardiac surgery unit | Assessment of each postoperative assessment by an HCP once per day during the hospitalization at the postoperative cardiac surgery unit  Intervention performed by HCP associated with these assessments | Adherence to best-practice guidelines: score on 2 ( /2)  Dichotomous variable:  0=the postoperative assessment by HCP was not performed at least once per day during the patient hospitalization  1=the postoperative assessment by BCP was performed once or more per day during the patient hospitalization  Dichotomous variable:  0=absence of intervention performed by HCP associated with these assessments  1=presence of intervention performed by HCP associated with these assessments  Descriptive data:  The number of postoperative assessments performed by HCP during the entire patient hospitalization at the postoperative cardiac surgery unit  The type of intervention performed by HCP |
| **Confounding variable related to patient characteristics** | | | |
| Sociodemographic characteristics | Sex and age of the patient during hospitalization at the postoperative cardiac surgery unit | Sex: male/female  Age: age of the patient with a minimum value of 18 years old | Categorical variable: Male, female  Discrete variable: age from 18 years old and above |
| Hospital length of stay | Number of days of the patient’s hospitalization after the cardiac surgery | Number of days of hospitalization, including the time spent in ICU and at the postoperative cardiac surgery unit between the admission at the ICU and the hospital discharge | Continuous variable: number of days (hours) of the patient’s hospitalization after the cardiac surgery |
| Comorbidities | Past medical history including medical condition and chronic illness | Charlson Comorbidity Index (CCI) (SD appendix) | Discrete variable: score on 24 based on the CCI |
| Type of cardiac surgery | The cardiac procedure performed by the operating team | Cardiac surgeries including coronary artery bypass graft (CABG), valve repair, and mixed surgery CABG/valve repairs | Categorical variable:  CABG  Valve repair  Mixed CABG/valve repair |
| Intensive care unit (ICU) length of stay | Total number of hours hospitalized at the ICU after the cardiac surgery | The number of hours spent at the ICU immediately after the cardiac surgery | Continuous variable: number of hours hospitalized at the ICU after the cardiac surgery |
| Prolonged mechanical ventilation (>24h) | Patient under mechanical ventilation beyond 24 hours after the cardiac surgery | The presence of a prolonged mechanical ventilation beyond 24 hours  Number of hours under mechanical ventilation beyond 24 hours | Dichotomous variable:  0=mechanical ventilation below 24 hours after the cardiac surgery  1= mechanical ventilation above 24 hours after the cardiac surgery  Continuous variable: the number of hours under mechanical ventilation beyond 24 hours |
| Failed extubation and reintubation after first extubation | Reintubation of the patient after the first extubation, during the hospitalization at the ICU after the cardiac surgery | Number of episodes of reintubation after the first extubation at the ICU after the cardiac surgery  Number of total hours under reintubation at the ICU after the cardiac surgery, including all episodes of reintubation | Discrete variable: number of episodes of reintubation after the first extubation at the ICU  Continuous variable: total number of hours under reintubation after the first extubation at the ICU |
| ICU readmission | Readmitted to the ICU after initial discharge from the ICU post cardiac surgery | Number of episodes of readmission to the ICU after initial discharge from the ICU  Total number of hours readmitted to the ICU after initial discharge from the ICU, all episodes included | Discrete variable: number of episodes of readmission to the ICU  Continuous variable: total number of hours readmitted to the ICU after the initial discharge from ICU |
| Postoperative cardiac surgery unit length of stay | Number of days hospitalized at the postoperative cardiac surgery unit | Number of days hospitalized at the postoperative cardiac surgery unit, from the admission to the unit, to the hospital discharge | Continuous variable: number of days hospitalized at the postoperative cardiac surgery unit (in hours) |
| 30-days readmission to the postoperative cardiac surgery unit after hospital discharge | Readmission to the postoperative cardiac surgery unit upon 30-days after hospital discharge following cardiac surgery | Presence of one or more episode of readmission to the postoperative cardiac surgery unit upon 30-days following cardiac surgery | Dichotomous variable:  0=absence of any episode of readmission to the postoperative cardiac surgery unit within 30-days of hospital discharge  1=presence of one or more episode of readmission to the postoperative cardiac surgery unit upon 30-days following hospital discharge after cardiac surgery |
| **Confounding variables related to interprofessional teams’ characteristics** | | | |
| Cardiac surgeon | The cardiac surgeon who performed the cardiac surgery, and was in charge of post-surgery patient care and follow-up | The cardiac surgeon who managed the care given by the interprofessional team in the ICU and at the postoperative cardiac surgery unit | Categorical variable: a confidential pairing system will be created to match each cardiac surgeon with a patient |
| Number of consultations outside the cardiac surgery team | Number of HCP with distinct expertise who performed at least one inpatient consultation during the hospitalization at the ICU and/or at the postoperative cardiac surgery unit | The total number of HCP with distinct expertise who performed at least one inpatient consultation  Type of expertise associated with the inpatient consultation | Discrete variable: total number of HCP  Categorical variable, Type of expertise:  Occupational therapist  Physical therapist  Respiratory therapist  Social worker  Skin and wound care therapist  Nutritionist  Medical speciality |
