# Supplementary material for: The level of adherence to best-practice guidelines by interprofessional teams with and without acute care nurse practitioners in cardiac surgery: A study protocol
Source: PLoS One. 2023 Mar 1;18(3):e0282467. doi: 10.1371/journal.pone.0282467 (PMC9976998; doi:10.1371/journal.pone.0282467)
Supplement: S4 Appendix — (DOCX) [file pone.0282467.s004.docx]

**SD Appendix**

**Charlson Comorbidity Index**

| **Variable** | Score |
| --- | --- |
| Myocardial infarction | 0 |
| Congestive heart failure | 2 |
| Peripheral vascular disease | 0 |
| Cerebrovascular disease | 0 |
| Dementia | 2 |
| Chronic pulmonary disease | 1 |
| Rheumatologic disease | 1 |
| Peptic ulcer disease | 0 |
| Mild liver disease | 2 |
| Diabetes without chronic complications | 0 |
| Diabetes with chronic complication | 1 |
| Hemiplegia/paraplegia | 2 |
| Renal disease | 1 |
| Any malignancy, including leukemia and lymphoma | 2 |
| Moderate or severe liver disease | 4 |
| Metastatic solid tumor | 6 |
| VIH/sida (AIDS/HIV | 4 |
| **Total** | 24 |
| Reference : Charlson, M. E., Pompei, P., Ales, K. L., & MacKenzie, C. R. (1987). A new method of classifying prognostic comorbidity in longitudinal studies: development and validation. *Journal of chronic diseases, 40*(5), 373-383; Quan, H., Li, B., Couris, C. M., Fushimi, K., Graham, P., Hider, P., . . . Sundararajan, V. (2011). Updating and validating the Charlson comorbidity index and score for risk adjustment in hospital discharge abstracts using data from 6 countries. *American journal of epidemiology, 173*(6), 76-682. doi:10.1093/aje/kwq433 | |
