# Supplementary material for: The level of adherence to best-practice guidelines by interprofessional teams with and without acute care nurse practitioners in cardiac surgery: A study protocol
Source: PLoS One. 2023 Mar 1;18(3):e0282467. doi: 10.1371/journal.pone.0282467 (PMC9976998; doi:10.1371/journal.pone.0282467)
Supplement: Attachment — Submitted filename: PONE-D-21-40085_R1.pdf [file pone.0282467.s006.pdf]

PLOS ONE

The level of adherence to best-practice guidelines by interprofessional teams with and without acute care nurse practitioners in cardiac surgery: a study protocol

--Manuscript Draft--

Manuscript Number:	PONE-D-21-40085R1
Article Type:	Study Protocol
Full Title:	The level of adherence to best-practice guidelines by interprofessional teams with and without acute care nurse practitioners in cardiac surgery: a study protocol
Short Title:	Adherence to best-practice guidelines by interprofessional teams with and without nurse practitioners
Corresponding Author:	Li-Anne Audet, MSc<br>McGill University Faculty of Medicine and Health Sciences<br>Montreal, Quebec CANADA
Keywords:	Acute care nurse practitioner, best-practice guidelines, cardiac surgery, interprofessional teams, quality of care
Abstract:	Background<br>Acute care nurse practitioners (ACNP) have been implemented in postoperative cardiac surgery settings and have shown to provide significant benefits to patients and organizations. To explain these associations, recent studies have suggested that ACNP increase the level of adherence to best-practice guidelines by interprofessional teams. However, it is unknown if interprofessional teams with ACNP are associated with higher levels of adherence to best-practice guidelines compared to interprofessional teams without ACNP. Furthermore, no extraction tool is available to measure the level of adherence to best-practice guidelines by interprofessional teams in postoperative cardiac surgery settings. This project aims to examine the level of adherence to best-practice guidelines of interprofessional teams with and without ACNP in a postoperative cardiac surgery setting in Québec, Canada.<br>Methods<br>A retrospective observational study will be conducted of 300 patients hospitalized between January 1, 2019 and January 31, 2020 in a postoperative cardiac surgery unit in Québec, Canada. Data will be collected from patient health records and electronic databases. An extraction tool will be developed based on systematic review of the literature, and will include best-practice guidelines and confounding variables related to patient and interprofessional teams' characteristics. Content and criterion validation, and a pilot-test will be conducted for the development of the tool. A multivariate linear regression model will be developed and adjusted for several confounding variables, in order to examine the association between interprofessional teams with and without ACNP, and the level of adherence to best-practice guidelines.<br>Discussion<br>This project represents the first study to examine the level of adherence to best-practice guidelines by interprofessional teams with and without ACNP in a postoperative cardiac surgery setting. The findings of this project will generate empirical data focusing on the contribution of ACNP within interprofessional teams, and ultimately enhance the delivery of high quality and evidence-based care for patients and families.
Order of Authors:	Li-Anne Audet, MSc
	Mélanie Lavoie-Tremblay, PhD
	Éric Tchouaket, PhD
	Kelley Kilpatrick, PhD
Response to Reviewers:	September 13th, 2022

Powered by Editorial Manager® and ProduXion Manager® from Aries Systems Corporation

	To the Editor of Plos One,<br><br>We thank the reviewer and the editor for their thoughtful comments and have summarized our modifications to the manuscript addressing their questions and suggestions in the table attached with our submission.<br><br>Please do not hesitate to contact us if you require any additional information or have any further questions.<br><br>We will look forward to hearing from you.<br><br>Sincerely,<br><br>Li-Anne Audet
**Additional Information:**	
**Question**	**Response**
**Financial Disclosure**<br><br>Enter a financial disclosure statement that describes the sources of funding for the work included in this submission. Review the submission guidelines for detailed requirements. View published research articles from *PLOS ONE* for specific examples.<br><br>This statement is required for submission and **will appear in the published article** if the submission is accepted. Please make sure it is accurate.	This project is funded by the Réseau de recherche en intervention en sciences infirmières du Québec (RRISIQ). LAA holds doctoral scholarships from the Fonds de recherche du Québec – santé as well as from the Québec Ministry of Education (Ministère de l'Éducation et de l'Enseignement Supérieur du Québec). ET holds a career award from the Fonds de recherche du Québec – Santé (Junior 2 Research Salary Award). KK holds a career award from the Fonds de recherche du Québec – Santé (Senior Research Salary Award) and holder of the Susan E. French Chair in Nursing Research and Innovative Practice. No funding sources were involved in the study conception and design, data collection, analysis, interpretation, or in the final decision to submit this manuscript for publication.

Powered by Editorial Manager® and ProduXion Manager® from Aries Systems Corporation

**Unfunded studies**<br>Enter: *The author(s) received no specific funding for this work.*<br><br>**Funded studies**<br>Enter a statement with the following details:<br>• Initials of the authors who received each award<br>• Grant numbers awarded to each author<br>• The full name of each funder<br>• URL of each funder website<br>• Have or will the sponsors or funders play any role in the study design, data collection (when applicable) and analysis, decision to publish, or preparation of the manuscript?<br>• **NO** - Include this sentence at the end of your statement: *The funders had and will not have a role in study design, data collection and analysis, decision to publish, or preparation of the manuscript.*<br>• **YES** - Specify the role(s) played.<br><br>* typeset	
**Competing Interests**<br><br>Use the instructions below to enter a competing interest statement for this submission. On behalf of all authors, disclose any competing interests that could be perceived to bias this work—acknowledging all financial support and any other relevant financial or non-financial competing interests.<br><br>This statement is required for submission and **will appear in the published article** if the submission is accepted. Please make sure it is accurate and that any funding sources listed in your Funding Information later in the submission form are also declared in your Financial Disclosure statement.<br><br>View published research articles from *PLOS ONE* for specific examples.	The authors have declared that non competing interests exist.

**NO authors have competing interests**<br><br>Enter: *The authors have declared that no competing interests exist.*<br><br>**Authors with competing interests**<br><br>Enter competing interest details beginning with this statement:<br><br>*I have read the journal's policy and the authors of this manuscript have the following competing interests: [insert competing interests here]*<br><br>* typeset	
**Ethics Statement**<br><br>Enter an ethics statement for this submission. This statement is required if the study involved:<br><br>• Human participants<br>• Human specimens or tissue<br>• Vertebrate animals or cephalopods<br>• Vertebrate embryos or tissues<br>• Field research<br><br>Write "N/A" if the submission does not require an ethics statement.<br><br>General guidance is provided below. Consult the submission guidelines for detailed instructions. **Make sure that all information entered here is included in the Methods section of the manuscript.**	Ethics approval:<br>Final REB Approval of a New Research Project<br>The level of adherence to best-practice guidelines of interprofessionnel teams with and without adult care nurse practitioner in postoperative cardiac surgery: a retrospective observational study (IPSSA chirurgiecardiaque / 2022-8094)<br>MUHC REB Co-Chair for the CTGQ panel: Me Marie Hirtle<br><br>Dear Dr. Lavoie-Tremblay,<br>Thank you for submitting your responses and corrections for the research project indicated above, as requested by the McGill University Health Centre (MUHC) Research Ethics Board (REB).<br>The MUHC REB, more precisely its Cells, Tissues, Genetics & Qualitative (CTGQ) research panel provided conditional approval for the research project after a delegated review provided by its member(s).<br>On 2021-09-15, a delegated review of your responses and corrections was provided by member(s) of the MUHC REB. The research project was found to meet scientific and ethical standards for conduct at the MUHC.<br><br>The following documents were approved or acknowledged by the MUHC REB:<br>Initial Submission Form (F11NIR-80464)<br>REB Conditions & PI Responses Form(s) (F20-82210, F20-83659) Documents utilisés pour le recrutement<br>(Poster_Fr_V2_2021-08-23_REB Approved.docx) [date : 2021-08-23]<br>(Poster_Eng_V2_2021-08-23_REB Approved.docx) [date : 2021-08-23] Protocole de recherche<br>(Research_proposal_Li-AnneAudet_V2_2021-08-23_Fr_REB Approved.docx) [date : 2021-08-23]<br>Documents utilisés pour le recrutement<br>(Recruitment_email_Fr_V2_2021-08-23_REB Approved.docx) [date : 2021-08-23]<br>(Recruitment_email_Eng_V2_2021-08-23_REB Approved.docx) [date : 2021-08- 23]<br>Questionnaire(s) et matériel de recherche<br>(Qualtrics_questionnaire_Fr_REB Approved.docx) [date : 2021-09-06]<br>(Qualtrics_questionnaire_Eng_REB Approved.docx) [date : 2021-09-06]<br>(Extraction_tool_Fr_REB Approved.docx)<br>(Extraction_tool_Eng_ REB Approved.docx)

Powered by Editorial Manager® and ProduXion Manager® from Aries Systems Corporation

<table>
<tr>
<td>
Format for specific study types

Human Subject Research (involving human participants and/or tissue)
• Give the name of the institutional review board or ethics committee that approved the study
• Include the approval number and/or a statement indicating approval of this research
• Indicate the form of consent obtained (written/oral) or the reason that consent was not obtained (e.g. the data were analyzed anonymously)

Animal Research (involving vertebrate animals, embryos or tissues)
• Provide the name of the Institutional Animal Care and Use Committee (IACUC) or other relevant ethics board that reviewed the study protocol, and indicate whether they approved this research or granted a formal waiver of ethical approval
• Include an approval number if one was obtained
• If the study involved non-human primates, add additional details about animal welfare and steps taken to ameliorate suffering
• If anesthesia, euthanasia, or any kind of animal sacrifice is part of the study, include briefly which substances and/or methods were applied

Field Research

Include the following details if this study involves the collection of plant, animal, or other materials from a natural setting:
• Field permit number
• Name of the institution or relevant body that granted permission
</td>
<td>
FIC approuvé(s) par le CÉR
(ICF_Fr_V2_2021-08-23_REB Approved.docx) [Date: 2021-08-23]
(ICF_Eng_V2_2021-08-23_REB Approved.docx) [Date: 2021-08-23]
This will be reported to the MUHC REB and will be entered accordingly into the minutes of the next CTGQ meeting. Please be advised that you may only initiate the study after all required reviews and decisions are received and documented and you have received the MUHC authorization letter.

Scientific approval:
Dear Li-Anne,
On behalf the IsoN Comprehensive Examination Committee, I am pleased to confirm that you successfully passed your PhD comprehensive examination on April 13, 2021 for your project entitled: Le niveau d’adhérence aux pratiques exemplaires des équipes interprofessionnelles avec et sans infirmières praticiennes spécialisées en soins aux adultes en contexte de chirurgie cardiaque : une étude observationnelle rétrospective.
Your comprehensive examination (both your written protocol and oral presentation of your proposed thesis research) demonstrated that you have sufficient strength in terms of scientific knowledge, originality of thought and the capacity for clear expression to continue your doctoral research.
The Comprehensive Examination Committee members (external examiners) were: Dr. Christine Maheu (Associate Professor, ISoN, Chair of the Comprehensive Examination Committee) and Dr. Andraea Van Hulst (Associate Professor, ISoN).
You have now advanced to the status of doctoral candidate. Congratulations on this important milestone, and we wish you success with the completion of your doctoral research.
Sincerely,
</td>
</tr>
<tr>
<td>
Data Availability

Authors are required to make the data underlying their research findings fully available, without restriction.

If the manuscript reports pilot or preliminary results, the data underlying those results must be made available at the time of publication. A Data Availability
</td>
<td>
N/A - the protocol does not report results
</td>
</tr>
</table>

Statement describing where the data can be found is required at submission. Your answers to this question constitute the Data Availability Statement and will be published in the article, if accepted. When legal or ethical requirements prohibit public data sharing, authors must indicate how researchers may obtain access to the data. See the PLOS Data Policy and FAQ for detailed information.<br><br>**Important:** Stating 'data available on request from the author' is not sufficient. If your data are only available upon request, select 'No' for the first question and explain your situation in the text box.<br><br>For protocols without pilot or preliminary data, authors are strongly encouraged to state how they plan to share research data from their study when it is completed or published.<br><br>Do the authors confirm that EITHER the protocol does not report results OR all data underlying the preliminary results will be made fully available without restriction?	
**Describe where the data may be found in full sentences. If you are copying our sample text, replace any instances of XXX with the appropriate details.**<br><br>• If **no pilot data are reported** in the manuscript, please enter the following: *No datasets were generated or analysed during the current study. All relevant data from this study will be made available upon study completion.*<br>• If the manuscript reports pilot data **held in a public repository**, include URLs, accession numbers or DOIs. If this information will only be available after acceptance, indicate this by ticking the box below. For example: *All XXX files are available from the XXX database*	No datasets were generated or analysed during the current study. All relevant data from this study will be made available upon study completion.

(accession number(s) XXX, XXX.).<br>• If the manuscript reports pilot data contained **within the manuscript and/or Supporting Information files**, enter the following:<br>*All relevant data are within the manuscript and its Supporting Information files.*<br>• If neither of these applies but you are able to provide **details of access elsewhere**, with or without limitations, please do so. For example:<br><br>*Data cannot be shared publicly because of [XXX]. Data are available from the XXX Institutional Data Access / Ethics Committee (contact via XXX) for researchers who meet the criteria for access to confidential data.*<br><br>*The data underlying the results presented in the study are available from (include the name of the third party and contact information or URL).*<br>• This text is appropriate if the data are owned by a third party and authors do not have permission to share the data.<br><br>For study protocols with data management or sharing plans, authors are encouraged to briefly describe in their Data Availability Statement how data they generate will be made accessible when the study is completed. For example:<br>• Deidentified research data will be made publicly available when the study is completed and published.<br><br>* typeset	
Additional data availability information:	

Powered by Editorial Manager® and ProduXion Manager® from Aries Systems Corporation

Manuscript Click here to access/download;Manuscript;Manuscript.docx

The level of adherence to best-practice guidelines by interprofessional teams with and without acute care nurse practitioners in cardiac surgery: a study protocol

Authors and affiliations: Li-Anne Audet, RN.[1], M.Sc, Mélanie Lavoie-Tremblay, RN.[2], Ph.D, Éric Tchouaket, Ph.D[3], Kelley Kilpatrick, RN., Ph.D[1,4,5]

[1] McGill University, Ingram School of Nursing, Faculty of Medicine and Health Sciences, 680 Sherbrooke West, Suite 1800, Montreal, QC, Canada, H3A2M7

[2] Université de Montréal, Faculté des sciences infirmières, Pavillon Marguerite-d'Youville, 2375 chemin de la Côte-Ste-Catherine, Montreal, QC, Canada, H3T1A8

[3] Université du Québec en Outaouais, Département des sciences infirmières, 5 rue Saint-Joseph, Saint-Jérôme, QC, Canada, J7Z0B7

[4] Centre intégré universitaire de santé et de services sociaux de l'Est-de-l'Île-de-Montréal-Hôpital Maisonneuve-Rosemont (CIUSSS-EMTL-HMR), 5415, boulevard de l'Assomption, Montreal, QC, Canada, H1T 2M4

[5] Susan E. French Chair in Nursing Research and Innovative Practice, McGill University, Ingram School of Nursing, Faculty of Medicine and Health Sciences, 680 Sherbrooke West, Suite 1800, Montreal, QC, Canada, H4A 2M7

Corresponding author: Li-Anne Audet, Ingram School of Nursing, McGill University, 680 Sherbrooke Street West, Montreal, QC, H3A2M7, Email address: li-anne.audet@mail.mcgill.ca

Word count: 5,721

The level of adherence to best-practice guidelines by interprofessional teams with and without acute care nurse practitioners in cardiac surgery: a study protocol

Short title

Adherence to best-practice guidelines by interprofessional teams with and without acute care nurse practitioners

Abstract

Background

Acute care nurse practitioners (ACNP) have been implemented in postoperative cardiac surgery settings and have shown to provide significant benefits to patients and organizations. To explain these associations, recent studies have suggested that ACNP increase the level of adherence to best-practice guidelines by interprofessional teams. However, it is unknown if interprofessional teams with ACNP are associated with higher levels of adherence to best-practice guidelines compared to interprofessional teams without ACNP. Furthermore, no extraction tool is available to measure the level of adherence to best-practice guidelines by interprofessional teams in postoperative cardiac surgery settings. This project aims to examine the level of adherence to best-practice guidelines of interprofessional teams with and without ACNP in a postoperative cardiac surgery setting in Québec, Canada.

Methods

A retrospective observational study will be conducted of 300 patients hospitalized between January 1, 2019 and January 31, 2020 in a postoperative cardiac surgery unit

in Québec, Canada. Data will be collected from patient health records and electronic databases. An extraction tool will be developed based on systematic review of the literature, and will include best-practice guidelines and confounding variables related to patient and interprofessional teams' characteristics. Content and criterion validation, and a pilot-test will be conducted for the development of the tool. A multivariate linear regression model will be developed and adjusted for several confounding variables, in order to examine the association between interprofessional teams with and without ACNP, and the level of adherence to best-practice guidelines.

Discussion

This project represents the first study to examine the level of adherence to best-practice guidelines by interprofessional teams with and without ACNP in a postoperative cardiac surgery setting. The findings of this project will generate empirical data focusing on the contribution of ACNP within interprofessional teams, and ultimately enhance the delivery of high quality and evidence-based care for patients and families.

Keywords

Acute care nurse practitioner, best-practice guidelines, cardiac surgery, interprofessional teams, quality of care

Background

Cardiac surgeries are one of the leading types of surgical procedures performed internationally. In 2017, approximately 1 million patients throughout the world underwent a cardiac surgery (1). Most cardiac surgeries are performed in developed countries (1). In 2018, the Society of Thoracic Surgeons (STS) reported 287,872 cardiac surgeries and procedures performed in the United States (2). In Canada, 42,989 coronary artery bypass grafts (CABG) and 7,186 valve repairs were performed between 2013 and 2016 (3, 4).

In the postoperative phase after the surgery, many patients are at high risk of developing adverse events and postoperative complications such as myocardial infarction and wound infection (5). These complications are associated with higher risk of mortality and development of comorbidities (e.g., heart failure), as well as a decrease in the well-being and quality of life of patients and families (6). For healthcare organizations, adverse events and postoperative complications are associated with a longer length of stay at the hospital, higher rates of readmission in intensive care units, higher rates of surgical re exploration, and an increase in cost (7, 8).

To prevent the risk of adverse events and postoperative complications for patients and families, international healthcare organizations and research teams have developed best-practice guidelines (7, 9). Best-practice guidelines are defined as evidence-based practice of care, and aim to ensure the performance of interprofessional teams, enhancing the quality and safety of the care given to patients and families. In postoperative cardiac surgery settings, best-practice guidelines related to the pharmacotherapy, laboratory tests, clinical indicators, and lifestyle promotion

(e.g., diet, cardiac rehabilitation) have been developed and implemented in healthcare centres (2).

A consensus in the literature supports the importance of a high level of adherence by interprofessional teams to best-practice guidelines, in order to ensure their efficacy (7, 10). Adherence to best-practice guidelines is defined as the achievement of the guideline, as well as the associated interventions performed by clinicians (11). In surgical settings (e.g., neurosurgery, orthopedic surgery) international research teams (12-15) have developed composite scores to examine the level of adherence to best-practice guidelines by interprofessional teams. Their findings suggested that a higher level of adherence is associated with a higher quality of care given to patients and families.

In postoperative cardiac surgery settings, recent studies (5, 16) identified a significant association between a high level of adherence to best-practice guidelines by interprofessional teams and a lower risk of postoperative complications and adverse events for patients. Other studies (5, 16, 17) have shown a significant association between a lower level of adherence to best-practice guidelines by interprofessional teams and a higher risk of postoperative complications and adverse events for patients. Larrazzet et al. (2014) conducted a longitudinal study of 144 patients who died after cardiac surgery. The authors identified a significant association between a lack of adherence to best-practice guidelines by interprofessional teams, and a higher risk of mortality, highlighting the importance of the high adherence to best-practice guidelines by interprofessional teams (17).

Internationally, there is variability in the scope of practice and level of autonomy of ACNPs (18,19). Recent studies have examined the practice of nurse practitioners (NP) within primary and acute care settings, and suggested that their practice could increase the level of adherence to best-practice guidelines by interprofessional teams (18, 19). The NP's support of the practice of different care providers in the team with a focus on best evidence is believed to underpin providers' adherence to best-practice guidelines focused on pharmacotherapy, clinical indicators, and lifestyle promotion. NPs also enhance collaboration and communication among teams' members and facilitate continuity of care, contributing to the higher adherence to best-practice guidelines by interprofessional teams (18, 20).

Based on the International Council of Nurses guidelines, the NP is an advanced practice nursing role, based on a graduate or postgraduate education and an in-depth nursing and medical expertise (21-23). In several acute care settings, including postoperative cardiac surgery, acute care nurse practitioners (ACNP) have been implemented in interprofessional teams and performed activities such as the clinical and psychosocial assessment of patients, the prescription and management of pharmacotherapy, laboratory tests and clinical interventions, lifestyle promotion, teaching of coping strategies, preparation for hospital discharge, and the management of consultations and external resources (24). International research teams (23, 25) have examined the practice of ACNPs within postoperative cardiac surgery settings. They identified significant associations between the ACNPs' practice and higher patient satisfaction, higher participation of patients to cardiac rehabilitation, and lower length of stay at the hospital after the cardiac surgery. However, other research teams (21, 26)

did not identify significant associations between ACNP practice and patient and organizational outcomes within postoperative cardiac surgery settings. More specifically, the authors suggested that the difficulty of recruiting participants, the high attrition rate, the small sample sizes, and the inability to adjust the data analysis for confounding variables related to the patient and interprofessional teams' characteristics were important limits contributing to the non-significant findings (21,26).

Systematic reviews of randomized controlled trials (RCT) (21, 23) have been conducted to understand the inconsistent findings in the current literature. These systematic reviews have identified three important limits contributing to the inconsistent findings of the current literature. Firstly, although several RCT of the current literature focus on the efficacy of ACNP for patients and healthcare organizations, less attention has been paid to the efficacy of these providers for interprofessional teams. The qualitative study of Reich et al. (2018) suggested that the implementation of ACNP within interprofessional teams increases the level of adherence of those teams to best-practice guidelines in postoperative cardiac surgery settings, and represents an underlying factor contributing to the efficacy of NP (19). However, these propositions have not been subjected to statistical validation, thus it is unknown if interprofessional teams with ACNP are associated with higher levels of adherence to best-practice guidelines, compared to interprofessional teams without ACNP. Moreover, until today, no validated extraction tool is available to measure the level of adherence to best-practice guidelines of interprofessional teams with and without ACNP in postoperative cardiac surgery settings.

Secondly, systematics reviews have highlighted the difficulty in adequality controlling confounding variables in the existing RCT (21, 23). Confounding variables represent important factors influencing the efficacy of ACNP on patient and organizational outcomes in postoperative cardiac surgery settings. Methodological limits of the existing RCT, such as the small sample size and high attrition rate, limit the capacity of research teams to develop robust multivariate statistical models adjusted for confounding variables related to patient, interprofessional teams, and organizational characteristics (21, 23). Interestingly, retrospective observational studies have shown to be a relevant alternative to examine the association between ACNP and patient and organizational outcomes in postoperative cardiac surgery settings. Existing studies (27-30) have used retrospective observational studies to gather a large cohort of patients, limit attrition, and develop statistical models adjusted for many confounding variables. These studies (6, 27-29) have identified significant associations between ACNP within interprofessional teams, and lower risks of mortality and decreased costs in postoperative cardiac surgery settings.

Thirdly, the majority of the RCT focus on ACNP practice within primary care settings (e.g., in-home care, rehabilitation clinic) after the cardiac surgery. Less attention has been paid to ACNP practice in acute care settings; however, within these settings, ACNP works in collaboration with cardiac surgeons, nursing teams, rehabilitation teams, social workers, and other members of the interprofessional teams to ensure the patient optimal recovery after the surgery and prevents the risk of adverse events and postoperative complications. Moreover, for patients and families, the acute care hospitalization after the surgery represents a crucial phase of their postoperative

recovery and is associated with several physical, psychosocial, and emotional stressors (30). Future studies are needed to substantiate the contribution of ACNP in interprofessional teams within acute care settings, and identify the benefits of their practice for patients, families, interprofessional teams, and healthcare organizations.

Consequently, the aim of this project is to examine the level of adherence to best-practice guidelines of interprofessional teams with and without ACNP in acute postoperative cardiac surgery settings in Québec, Canada.

Study hypothesis and objectives

The hypothesis of this study is:

H1: This study will verify if interprofessional teams with ACNP are associated with a higher level of adherence to best-practice guidelines compared to interprofessional teams without ACNP, after controlling for the patient and interprofessional team characteristics.

The objectives of this study are as follows:

1. Develop and pilot-test an extraction tool to measure the level of adherence to best-practice guidelines of interprofessional teams within postoperative cardiac surgery setting.
2. Describe the patient and interprofessional teams' characteristics, as well as the level of adherence to best-practice guidelines, of patients under the care of interprofessional teams with and without ACNP.
3. Examine the association between interprofessional teams with ACNP and the level of adherence to best-practice guidelines, compared to interprofessional

teams without ACNP, after adjusting patient and interprofessional team characteristics in postoperative cardiac surgery setting.

Methods and design

Study design

A retrospective observational study (31, 32) will be conducted. De-identified data will be extracted from the UHC data warehouse, and the patient health records to assemble the retrospective cohort of patients and conduct the data collection. Ethics approval was obtained from the McGill University Health Centre Research Ethics Board on September 15, 2021 (IPSSA chirurgie cardiaque/2022-8094). This project uses the guidelines from *Strengthening the Reporting of Observational Studies in Epidemiology (STROBE) Statement: Guidelines for Reporting Observational Studies* (32) were used for this project (SA appendix).

Study setting

This study will be conducted at a University healthcare centre (UHC) in Québec, Canada. Annually, this healthcare centre performs approximately 1,000 cardiac surgeries and has a 36-bed postoperative cardiac surgery unit.

After cardiac surgery, patients are admitted to the intensive care unit (ICU), and are under the care of ICU teams. At the time of data collection, the ICU teams in Québec, Canada do not include ACNP. On average, patients are hospitalized for 24 hours in the ICU before being transferred to the postoperative cardiac surgery unit. Patients readmitted to the ICU are under the care of ICU teams.

Upon admission to the postoperative cardiac surgery unit, patients are assigned to the care of interprofessional teams with or without ACNP. Team assignment depends

on the current workload of each team, and the availability of the beds in the unit. Interprofessional teams with ACNPs are assigned 16 of the unit’s beds (44%) and interprofessional teams without ACNP are assigned 20 of the unit’s beds (56%). Patients are followed by their respective teams from their admission to the cardiac surgery unit, until the hospital discharge or death. On average, the length of stay at the hospital after the surgery ranges from 9 to 11 days (33).

Interprofessional teams with and without ACNP include the following: cardiac surgeons and residents nursing teams, skin care and wound therapists, physiotherapists, nutritionists, respiratory therapists, social workers, and medical and other specialists. Interprofessional teams with and without ACNP ensure the daily follow-up of patients, the assessment of the patient's clinical and psychosocial condition, the management of pharmacotherapy, clinical intervention, laboratory tests, lifestyle promotion, and the preparation of patients upon hospital discharge.

The practice of acute care nurse practitioners in postoperative cardiac surgery unit

Since 2017, seven ACNPs practice in postoperative cardiac surgery, based on a monthly rotation system. Two ACNP simultaneously practice within interprofessional teams and collaborate with other clinicians to ensure the daily follow-up of the patients after cardiac surgery. ACNP practice includes the assessment of the clinical and psychosocial condition of the patient and families, the management of the pharmacotherapy, clinical interventions, and laboratory tests. The ACNP also teaches coping strategies, lifestyle promotion, cardiac rehabilitation, preparation of the patients and families for the in-home recovery after discharge. The ACNP supports the

preparation for hospital discharge (e.g., prescription of the patient medication after discharge, summary for the family physician, etc.) (34).

Study population and sample

A dynamic cohort of patients will be assembled from hospitalized patients at the postoperative cardiac surgery unit between January 1, 2019, and January 31, 2020. This timeframe will avoid the recruitment of patients during the COVID-19 pandemic, a window of time during which the practice of ACNP within the unit was inconsistent. Patients will be selected, based on three inclusion criteria: 1) patient admitted for a diagnosis of CABG and/or valve repair, 2) patient age is over 18 years old, and 3) patient has been hospitalized for at least 24 hours in the postoperative cardiac surgery unit. The minimum 24-hour criteria will allow our team to examine the hospitalization of the patient in the postoperative cardiac surgery unit, which is the setting where ACNPs practice in Québec (35). Patients will be identified from the electronic databases of the UHC and the diagnostic codes of the Canadian Institute for Health Information (36) (SB appendix).

A sample size calculation was conducted in G*power (37). This project will include 15 variables, including one independent variable, one dependent variable, and 13 confounding variables. The variables selected for this project are described in the next section. To perform one multivariate linear regression model and detect an effect size of f^2=0.15 with a power of 0.80 and a standard error of 5%, a minimum sample size of 183 patients was estimated.

A total of 300 patients will be selected, based on the sample size calculation and an over-estimation of at least 20%, to consider the risk of error from the presence of

missing data and incomplete patient health records (38, 39). A systematic random selection of 150 patients under the care of interprofessional teams with ACNP will be conducted and matched with 150 patients under the care of interprofessional teams without ACNP. Three criteria (40-43) will be used to match each pair (1:1): 1) age (i.e., a five-year gap will be tolerated) 2) sex, and 3) type of cardiac surgery (i.e., CABG, valve repair, or CABG/valve repair).

Patients will be followed from their admission to the ICU after the cardiac surgery, until the occurrence of the following events, whichever occurs first: 1) discharge from the hospital, 2) death, or 3) the cumulation of 14-days in hospital after the surgery. The 14-days' timeframe will allow our team to capture the acute phase of the patient hospitalization after the cardiac surgery (33). The total length of stay for patients hospitalized longer than 14-days will be measured.

Variables under study

A systematic review of RCTs (24) and an extensive search in the literature and international healthcare organizations (e.g., American Heart Association, Society of Thoracic Surgeons) was conducted to retrieve best-practice guidelines for interprofessional teams in postoperative cardiac surgery settings, and confounding variables related to patient and interprofessional teams' characteristics. A total of 12 best-practice guidelines and 13 confounding variables were identified and presented in Figure 1. The operationalization of each variable is presented in SC appendix.

Independent variable: interprofessional teams with and without acute care nurse practitioner

A dichotomous variable will be created to measure the inclusion of at least one ACNP within the interprofessional team. For patients under the care of an interprofessional team with ACNP, an additional descriptive variable will measure the number of days where the ACNP was implicated in the daily follow-up of the patient, on the entire hospitalization at the postoperative cardiac surgery unit.

Dependant variable: the level of adherence to best-practice guidelines by interprofessional teams

A composite score will be developed to measure the level of adherence to best-practice guidelines by interprofessional teams. An individual composite score will be calculated per patient per day of hospitalization at the postoperative cardiac surgery unit. A total of 12 best-practice guidelines will be included in the composite score (Figure 1). Each best-practice guideline will be scored on two points based on the following: 1) the achievement of the guideline recommendation (e.g., prescription of beta blocker) and 2) the associated interventions performed by clinicians (e.g., follow-up of the blood pressure). All best-practice guidelines are divided into three categories: 1) pharmacotherapy (n=4), 2) laboratory tests (n=4), and 3) postoperative assessment (n=4).

Four best-practice guidelines are included in the category of pharmacotherapy. These guidelines are the prescription and monitoring of medications including: 1) anticoagulants, 2) beta blockers, 3) lipid-lowering agents, and 4) anti-platelets, during hospitalization and at hospital discharge of the patient. For each best-practice guideline, two additional descriptive variables will be collected, including the type of medication

prescribed and the presence of a contraindication, which precludes the possibility of achieving the guideline recommendation (e.g., allergy).

Four best-practice guidelines on laboratory tests are included, recommending the prescription and monitoring of the following: 1) potassium (K+), 2) magnesium (Mg+), 3) serum glucose, and 4) international normalized ratio (INR) by interprofessional teams during the patient's hospitalization in the postoperative cardiac surgery unit. The achievement of each best-practice guideline recommendation will be confirmed if the laboratory result falls under the normal range supported by the current literature. An additional variable called "no INR needed" will be added specifically for the best-practice guideline related to the prescription and management of INR, to take into consideration patient who don't need a strict INR follow-up (e.g., administration of direct acting oral anticoagulant (DAOC)). A pilot test will be performed to assess the characteristics of the patients hospitalized at the post-operative cardiac surgery unit and the feasibility of the data collection. The following three additional descriptive variables will be measured for each guideline: 1) number of prescriptions requested by interprofessional teams, 2) proportion of abnormal values below or above normal range, and 3) average of all values of laboratory tests.

Four best-practice guidelines are included in the category of the postoperative assessment performed by interprofessional teams. A daily assessment of the patient's level of pain, surgical wound(s), nutrition, and mobilization will be measured. For each best-practice guideline recommendation, the achievement of the guideline will be confirmed if at least one postoperative assessment per day is performed by the ACNP or other members of the interprofessional teams during the patient's hospitalization in

the postoperative cardiac surgery unit. Two additional descriptive variables will be measured for each best-practice guideline, including: 1) the total number of postoperative assessments performed by interprofessional teams during the hospitalization, and 2) the type of associated interventions conducted by clinicians (e.g., non-pharmacological, consultation, etc.).

Confounding variables related to patient characteristics

Eleven confounding variables related to patient characteristics will be measured. For each patient, sociodemographic characteristics (e.g., sex, age) will be measured. The total length of stay at the hospital will be measured, which includes the stay in the ICU and postoperative cardiac surgery unit. The type of cardiac surgery will be collected from a categorical variable with three categories: 1) CABG, 2) valve repair, and 3) CABG/valve repairs. Comorbidities will be measured and operationalized from the Charlson Comorbidity Index (CCI) (44, 45). A total score of 24 points will be calculated for each patient based on the 17 clinical conditions included in the CCI (SD appendix). The length of stay in the postoperative cardiac surgery unit will be collected for each patient. The hospital 30-days readmission at the postoperative cardiac surgery unit after discharge will be measured for each patient from a dichotomous variable.

Four confounding variables related to the patient characteristics in the ICU setting will be measured. The total number of hours hospitalized in the ICU will be measured from the time (in hours) of admission to the ICU after the surgery and the time (in hours) of discharge of the patient. The postoperative prolonged mechanical ventilation will be measured from a dichotomous variable. A prolonged mechanical ventilation will be confirmed if the time under mechanical ventilation exceeds 24 hours.

The failed extubation of the patient and reintubation after first extubation will be measured with a dichotomous variable. The number of episodes of ICU readmission after initial discharge will be measured for each patient.

Confounding variables related to interprofessional teams' characteristics

Two confounding variables related to interprofessional teams' characteristics will be measured. First, the cardiac surgeon who performed the surgery and ensured the follow-up of the patient's recovery will be noted. A confidential code will be created for each surgeon practising in the UHC. Second, a discrete variable will be created to measure the number of consultations conducted by clinicians from different expertises (e.g., nutrition, rehabilitation team) during the patient's hospitalization in the postoperative cardiac surgery unit. A categorical descriptive variable will be created to measure each clinician's expertise, including the following: 1) rehabilitation team (e.g., physical therapist, occupational therapist), 2) respiratory therapist, 3) social worker, 4) skin and wound care therapist, 5) nutritionist, and 6) speciality consultation (e.g., internal medicine, nephrology).

Data collection

An extraction tool will be developed and pilot-tested, based on the best-practice guidelines and the confounding variables related to the patient and interprofessional team characteristics (Figure 1). One extraction will be completed per patient per day during the hospitalization in the postoperative cardiac surgery unit, by the first author or a research assistant. The development of the extraction tool followed the Consensus-based Standards for the selection of health measurement instrument (COSMIN)

guidelines (46) and will include three stages: the content validation; the criteria validation; and the pilot-test.

Data collection for the content validation

Content validation will be performed following two steps (46). First, an expert committee will be recruited from clinicians, managers, and researchers who have expertise in postoperative cardiac surgery. The identification and recruitment of experts will be conducted by our research team and will include experts in Canada. A total of 10 experts will be recruited, including at least the following five: 1) an ACNP who worked a minimum of one year within a postoperative cardiac surgery unit outside of our associated UHC, 2) a cardiac surgeon or fellow in cardiac surgery who worked at least two years in an acute care centre outside of our associated UHC, 3) a nurse manager who manages, at least one year, a postoperative cardiac surgery unit, within our associated UHC, 4) a healthcare professional who worked in the data warehouse of our associated UHC for at least one year, and 5) a researcher in nursing or healthcare sciences with an expertise in measurement instruments and/or cardiac surgery.

Second, an electronic survey (47) will be created and composed of all items of the extraction tool (best-practice guidelines and confounding variables). For each item, experts will assess its relevance using a 5-points Likert scale (48, 49). Additional spaces for qualitative comments and suggestions will be included in the survey. Two rounds of revision are planned. After the first round, our team will update the extraction tool and revise any unclear items based on the experts' recommendations, and an updated version will be resubmitted to the expert committee.

Data collection for the criterion validation

The criterion validation of the extraction tool will follow three consecutive steps. Firstly, the best-practice guidelines of The Society of Thoracic Surgeons (STS) have been selected as the gold standard of comparison for this study. For many years, the STS has been a leading healthcare organization in the development and validation of best-practice guidelines and performance measures in the care of patients in cardiac surgery settings (10). In 2007, the STS developed and validated 21 performance measures for the delivery of high quality of care for patients who underwent CABG, valve repair, and CABG/valve repairs (50, 51). For this project, five performance measures were selected, including: 1) prolonged mechanical ventilation higher than 24 hours in the ICU; 2) a 30-day readmission rate at the postoperative cardiac surgery unit after hospital discharge; and prescription of the 3) anti-platelet, 4) beta blocker, 5) and lipid-lowering agent, during the hospitalization at the postoperative cardiac surgery unit and at discharge. Each performance measure is operationalized as the proportion of patients who achieved the outcome (e.g., proportion of patients who were under prolonged mechanical ventilation) over the total sample size (46, 47).

Secondly, 30 patient health records will be reviewed independently by two reviewers on two occasions, including the first author and a research assistant. For the first round of revision, the patient's health record will be reviewed with the extraction tool. For the second round of revision, the records will be reviewed with the five performance measures from the STS.

Data collection for the pilot-test

A pilot-test will be conducted to assess the feasibility of the tool, standardize data collection among reviewers, examine the quality and accessibility of the retrospective

data, and update the tool if needed. Four iterative steps will be included in the pilot-test (52-55). First, two reviewers will conduct the pilot-test and data collection. Training sessions and documentation will be given to the reviewers.

Secondly, 30 patient health records from hospitalized patients in the postoperative cardiac surgery unit between January 1, 2019, and January 31, 2020, will be randomly selected. These patients will not be included in the retrospective cohort. Data will be collected by the two reviewers with the extraction tool. Inter-rater reliability will be assessed and a Cohen kappa higher than 0.60 will be targeted (55).

Thirdly, a triangulation of the available data will be performed to assess the quality and accessibility of the data. Multiple data sources will be screened between the electronic databases and patient health records. Items from the extraction tool with a high quantity of missing data or low quality of available data will be modified or removed.

Finally, frequent meetings among all research team members will be conducted to discuss divergences, reinforce convergences, and establish a consensus. The extraction tool will be updated after the pilot-test if needed (52).

During data collection from the retrospective cohort of patients, an additional 30 patient health records will be reviewed by two independent reviewers to ensure the standardization of the data collection (49, 50). Inter-rater reliability will be assessed and a Cohen Kappa value higher than 0.60 will be targeted (55).

Data analysis

Statistical analysis will be conducted to meet objectives one, two and three. The unit of observation will be the patient-level and the unit of analysis will be the interprofessional team-level.

Objective 1

The content validation of the extraction tool will include two rounds of revisions, with additional rounds if needed (56, 57). For each round of revision, a Fleiss Kappa will be calculated and a value between 0.60 and 0.80 will be targeted (48, 49). The content validity index (CVI) of each individual item of the extraction tool will be calculated from the proportion of experts who rated a score of 4 or 5 on the Likert scale (58). A CVI of 0.80 will be targeted for each item and items with a value below 0.80 will be removed or modified. Then, the proportion of items with a CVI value above 0.80 will be calculated to determine the overall CVI of the extraction tool. A value higher than 0.80 for the overall CVI of the extraction tool will be targeted (46). Qualitative comments and recommendations by experts will be analyzed by content analysis (59).

The criteria validation will be assessed using 30 patient health records. Five performance measures developed by the STS will serve as the gold standard for the comparison (10). First, the proportion of patients who achieved each performance measure over the total sample size (n=30) will be calculated, from the measures collected with the gold standard, as well as the measures collected with the extraction tool. Second, Spearman correlation will be calculated to assess the correlation between the proportions obtained from the measures of the gold standard, and the proportions obtained from the measures of the extraction tool. Values of Spearman correlation higher than 0.70 will be targeted (48, 49).

Objective 2

Descriptive analysis will be performed to assess the level of adherence by interprofessional teams with and without ACNP, as well as the confounding variables for patients under the care of both teams (60). An analysis of missing data will be conducted to examine the quality and distribution of missing data within patient health records and electronic databases. Team meetings and consultations with clinicians and statisticians will be held to discuss and identify potential causes of missing data, as well as to conduct a statistical analysis to manage them (61).

A composite score will be developed to measure the level of adherence to best-practice guidelines by interprofessional teams with and without ACNP. A composite score per patient per day of hospitalization in postoperative cardiac surgery will be developed and based on four consecutive steps. Firstly, for each best-practice guideline, a score on a scale of two will be created and points will be attributed for: 1) the achievement of the best-practice guideline recommendation, and 2) the associated intervention performed by clinicians. Points will be given based on the information contained in the patient health record and electronic databases. A missed intervention will be considered as a lack of adherence to best-practice guidelines, and no point will be given.

Secondly, all best-practice guidelines are divided into three categories: 1) pharmacotherapy, 2) laboratory tests, and 3) postoperative assessments. The sum of the individual score of each best-practice guideline will be calculated for each category. More specifically, each category includes four best-practice guidelines, and will be based on a total score ranging from zero to eight.

Thirdly, an overall composite score will be calculated from the sum of all the scores of the three categories, ranging from zero to 24. The overall composite score will be transformed into a value in percentage (0 to 100%) and an increase of the score will be associated with an increase of the level of adherence to best-practice guidelines by interprofessional teams. An overall score will be calculated per patient per each day hospitalized in the postoperative cardiac surgery unit.

Finally, an average composite score will be calculated from the average of all overall scores during the patient's hospitalization at the postoperative cardiac surgery unit. An average composite score will be calculated for all patients included in the retrospective cohort. A graphic illustration will be created from all average scores to visualize the tendency and distribution of the scores of the cohort.

Objective 3

Bivariate analysis will be conducted, and the statistical significance will be based on a *p*-value of 0.05 (60). The Bonferroni correction will be applied for the examination of each confounding variable related to the patient and interprofessional teams' characteristics (62).

A multivariate linear regression model will be developed to examine the association between interprofessional teams with and without ACNP and the level of adherence to best-practice guidelines (63-65). An examination of the distribution of the data will be performed. In the case of an abnormal distribution of the data, a transformation will be conducted to adjust the statistical model based on the observed data. The independent variables will include the interprofessional teams with and without ACNP, as well confounding variables. A correlation matrix will be developed,

and tolerance value lower than 0.2 and a variance inflation factor (VIF) value higher than 5 will be used to identify multicollinearity between independent variables (63-65). Independent variables with multicollinearity will be removed one at the time and a new regression will be calculated. The dependent variable of the regression model will be the average composite score (%) of the level of adherence to best-practice guidelines. Statistical significance will be based on a *p*-value of 0.05. Sensitivity analysis will be conducted based on a selection of confounding variables related to the characteristics of the patient (e.g., length of stay, ICU readmission). The selection of these confounding variables will be based on the observed data and descriptive analysis. The residual analysis and the analysis of the R^2 will be conducted to assess the goodness-of-fit of the multivariate linear regression model.

Interpretation of the findings

Consultations with clinicians and managers practising in postoperative cardiac surgery settings in Québec and Canada will be conducted to gain an in-depth understanding of the findings. These consultations will allow our team to explore potential underlying factors and mechanisms which could influence the level of adherence to best-practice guidelines by interprofessional teams with and without ACNP. Field notes will be taken during these consultations and analyzed using content analysis (45).

Ethical considerations

Ethics approval was obtained prior to the beginning of the study. No identifying information will be collected during the content validation with the expert committee and the data collection within electronic databases and patient health records. A confidential pairing system will be created to match the patient identification with a random number

(66). The database of the project will be kept in a secure server, protected by a confidential numeric code known only by the research team. During the dissemination of the findings, data will be shared in an aggregated form and the identity of the UHC, as well as the identity of the patients and clinicians, will remain confidential.

Discussion

ACNPs hold an important place within interprofessional teams caring for patients and families following cardiac surgery. For patients and families, ACNPs contribute to the management of pharmacotherapy, clinical and psychosocial conditions, as well as lifestyle promotion and teaching of coping strategies. For interprofessional teams, ACNPs enhance the collaboration and communication among team members and reinforce continuity of care. Until now studies examined the efficacy of ACNP on patient and organization outcomes within postoperative cardiac surgery settings; however, less attention has been paid to underlying factors contributing to the effect of ACNPs on these outcomes.

The findings of this study will further our understanding of the contributions of ACNPs in interprofessional teams practising in postoperative cardiac surgery settings in Québec, Canada and internationally. This project will generate empirical data to support and document the practice of ACNPs within interprofessional teams in four important ways. Firstly, as part of this project, a systematic review of randomized controlled trials was published to retrieve the current literature on the efficacy of ACNP and advanced practice nursing roles in postoperative cardiac surgery settings, and to examine their adherence to best-practice guidelines (40). Secondly, an extraction tool focusing on the level of adherence to best-practice guidelines by interprofessional

teams in postoperative cardiac surgery will be available for the field of cardiac surgery research and the scientific community more broadly. Thirdly, for patients under the care of interprofessional teams with and without ACNP, the findings will provide a description of the patient and interprofessional team characteristics as well as the adherence to best-practice guidelines. Finally, this study will examine the effect of ACNPs on the adherence to best-practice guidelines in settings where ACNPs are incorporated within inter-professional teams, and provide empirical data for those settings looking to introduce such roles in a postoperative cardiac surgery setting.

Funding

This project is funded by the Réseau de recherche en intervention en sciences infirmières du Québec (RRISIQ). Li-Anne Audet holds doctoral scholarships from the Fonds de recherche du Québec – santé as well as from the Québec Ministry of Education (Ministère de l’Éducation et de l’Enseignement Supérieur du Québec). Éric Tchouaket holds a career award from the Fonds de recherche du Québec – Santé (Junior 2 Research Salary Award). Kelley Kilpatrick holds a career award from the Fonds de recherche du Québec – Santé (Senior Research Salary Award) and holder of the Susan E. French Chair in Nursing Research and Innovative Practice. No funding sources were involved in the study conception and design, data collection, analysis, interpretation, or in the final decision to submit this manuscript for publication.

Competing interest

None to declare.

Acknowledgments

The authors would like to acknowledge the comments received from Christine Maheu, Andraea Van Hulst, and Alain Biron.

Author Contributions

Conceptualization: Li-Anne Audet, Mélanie Lavoie-Tremblay, Éric Tchouaket, Kelley Kilpatrick

Methodology: Li-Anne Audet, Mélanie Lavoie-Tremblay, Éric Tchouaket, Kelley Kilpatrick

Supervision: Mélanie Lavoie-Tremblay, Éric Tchouaket, Kelley Kilpatrick

Writing – original draft: Li-Anne Audet

Writing – review & editing: Mélanie Lavoie-Tremblay, Éric Tchouaket, Kelley Kilpatrick

References

1. Caplan LR. Primer on cerebrovascular diseases. London, United Kingdom: Academic Press is an imprint of Elsevier; 2017.

2. Bowdish ME, D'Agostino RS, Thourani VH, Desai N, Shahian DM, Fernandez FG, et al. The Society of Thoracic Surgeons Adult Cardiac Surgery Database: 2020 Update on Outcomes and Research. The Annals of thoracic surgery. 2020;109(6):1646-55.

3. Philippon F, Racine N, Labrecque N, Paradis M-J. Réseau québécois de cardiologie tertiaire. Rapport d’activité 2010-2013. Québec, QC; 2013.

4. Canadian Institute of Health Information. Cardiac Care Quality Indicators Report. Ottawa, ON: Canadian Institute for Health Information
; 2017.

5. Bourgon Labelle J, Audet L, Farand P, Rochefort C. Are hospital nurse staffing practices associated with postoperative cardiac events and death? A systematic review. PLOS ONE [Internet]. 2019; 14(10).

6. Lane-Fall MB, Ramaswamy TS, Brown SES, He X, Gutsche JT, Fleisher LA, et al. Structural, Nursing, and Physician Characteristics and 30-Day Mortality for Patients Undergoing Cardiac Surgery in Pennsylvania. Critical care medicine. 2017;45(9):1472-80.

7. Benjamin JE, Virani SS, Callaway WC, Chamberlain MA, Chang RA, Cheng ES, et al. Heart Disease and Stroke Statistics—2018 Update: A Report From the American Heart Association. Circulation. 2019;137(12):e67-e492.

8. Goldfarb M, Bendayan M, Rudski LG, Morin JF, Langlois Y, Ma F, et al. Cost of Cardiac Surgery in Frail Compared With Nonfrail Older Adults. The Canadian journal of cardiology. 2017;33(8):1020-6.

9. McConnell G, Woltz P, Bradford WT, Ledford JE, Williams JB. Enhanced recovery after cardiac surgery program to improve patient outcomes. Nursing. 2018;48(11):24-31.

10. The Society of Thoracic Surgeons. Performance Measures 2019 [internet],Chicago (USA):The Society of Thoracic Surgeons; 2019 [updated 2019; cited 2021 Aug 24]. Available from: https://www.sts.org/quality-safety/performance-measures].

11. Khalil V, Danninger M, Wang W, Khalil H. An audit of adherence to heart failure guidelines in an Australian hospital: A pharmacist perspective. Journal of Evaluation in Clinical Practice. 2017;23(6):1195-202.

12. Burnier M, Egan BM. Adherence in Hypertension: A Review of Prevalence, Risk Factors, Impact, and Management. Circulation Research. 2019;124(7):1124-40.

13. Chan W, Chen A, Tiao D, Selinger C, Leong R. Medication adherence in inflammatory bowel disease. Intestinal Research [Internet]. 2017; 15(4):434-45.

14. López-Campos JL, Quintana Gallego E, Carrasco Hernández L. Status of and strategies for improving adherence to COPD treatment. International Journal of COPD [Internet]. 2019; 14:1503-15.

15. Moon SJ, Lee WY, Hwang JS, Hong YP, Morisky DE. Accuracy of a screening tool for medication adherence: A systematic review and meta-analysis of the Morisky Medication Adherence Scale-8. PloS one. 2017;12(11):e0187139.

16. Witwer M, Dobbins G, Uher C, McFarren M. Auditing CAUTI Best Practices Bundle Adherence in a 24-bed Neurovascular/Cardiovascular/Intensive Care Unit. AJIC: American Journal of Infection Control. 2019;47(6 Supplement):S30.

17. Larrazet F, Folliguet T, Laborde F, Bachet J, Hervé C. Mortality meetings: ethical considerations and adherence to evidence-based practice from 6 years of experience in a mixed cardiac department. European journal of cardio-thoracic surgery : official journal of the European Association for Cardio-thoracic Surgery. 2011;40(5):1039-45.

18. Norful AA, Ye S, Van der-Biezen M, Poghosyan L. Nurse Practitioner-Physician Comanagement of Patients in Primary Care. Policy, politics & nursing practice. 2018:1527154418815024.

19. Reich E, Then K, Rankin J. Barriers to Clinical Practice Guideline Implementation for Septic Patients in the Emergency Department. Journal of Emergency Nursing. 2018;44(6):552-62.

20. Kleinpell. Outcome assessment in advanced practice nursing. New York, NY: Springer Publishing Company, LLC; 2017.

21. Smigorowsky MJ, Sebastianski M, Sean McMurtry M, Tsuyuki RT, Norris CM. Outcomes of nurse practitioner-led care in patients with cardiovascular disease: A systematic review and meta-analysis. Journal of advanced nursing. 2020;76(1):81-95.

22. Yang BK, Johantgen ME, Trinkoff AM, Idzik SR, Wince J, Tomlinson C. State Nurse Practitioner Practice Regulations and U.S. Health Care Delivery Outcomes: A Systematic Review. Medical care research and review : MCRR. 2020:1077558719901216.

23. Mares M, McNally S, Fernandez R. Effectiveness of nurse-led cardiac rehabilitation programs following coronary artery bypass graft surgery: a systematic review. 2018;16(12):2304-29.

24. Audet L, Paquette L, Bordeleau S, Lavoie-Tremblay M, Kilpatrick K. The association between advanced practice nursing roles and outcomes in adults following cardiac surgery: A systematic review of randomized controlled trials. International journal of Nursing Studies. 2021.

25. Kwak C, Ko Y. Clinical Nurse Specialist–Led Implementation of an Early Discharge Protocol After Cardiac Surgery. Clin Nurse Spec. 2019;33(4):184-90.

26. Doyle L. A systematic review to enquire is Advanced Nurse Practitioner care more effectivve for the cardiac surgery patient, compared to standard care. European Journal of Cardiovascular Nursing. 2015;1:90.

27. Hall MH, Esposito RA, Jahn L, Blenderman R, Hartman AR, Pekmezaris R, et al. Cardiac surgery nurse practitioner home visits prevent coronary artery bypass graft readmissions. Annals of Thoracic Surgery. 2014;97(5):1488-95.

28. Southey D, Mishra PK, Nevill A, Aktuerk D, Luckraz H. Continuity of care by cardiothoracic nurse practitioners: Impact on outcome. Asian Cardiovascular and Thoracic Annals. 2014;22(8):944-7.

29. Skinner H, Skoyles J, Redfearn S, Jutley R, Mitchell I, Richens D. Advanced care nurse practitioners can safely provide sole resident cover for level three patients: impact

on outcomes, cost and work patterns in a cardiac surgery programme. European Journal Of Cardio-Thoracic Surgery. 2013;43(1):19-22.

30. Bourgon Labelle J, Farand P, Vincelette C, Dumont M, Le Blanc M, Rochefort CM. Validation of an algorithm based on administrative data to detect new onset of atrial fibrillation after cardiac surgery. BMC Medical Research Methodology. 2020;20(1).

31. Needleman J, Buerhaus P, Pankratz VS, Leibson CL, Stevens SR, Harris M. Nurse staffing and inpatient hospital mortality. New England Journal of Medicine. 2011;364(11):1037-45.

32. von Elm E, Altman DG, Egger M, Pocock SJ, Gøtzsche PC, Vandenbroucke JP. The Strengthening the Reporting of Observational Studies in Epidemiology (STROBE) Statement: guidelines for reporting observational studies. International Journal Of Surgery (London, England). 2014;12(12):1495-9.

33. Bourgon Labelle J. Associations entre les pratiques quotidiennes de gestion du personnel de soins et l'incidence de la fibrillation auriculaire postopératoire en chirurgie cardiaque. Savoirs UdeS: Université de Sherbrooke; 2020.

34. Ordre des infirmières et infirmiers du Québec. Encadrement de la pratique des IPS Montréal [internet]. Québec (Can):Ordre des infirmières et infirmiers du Québec;2021 [updated 2021 cited 2021 Aug 24]. Available from: https://www.oiiq.org/en/pratique-professionnelle/pratique-avancee/ips/encadrement-de-la-pratique-des-ips

35. Hoffman LA, Guttendorf J. Integrating Nurse Practitioners into the Critical Care Team. Critical Care Alert. 2014;21(12).

36. Canadian Institute of Health Information. Canadian Coding Standards for ICD-10-CA and CCI [internet].Ottawa (Can):Canadian Institute of Health Information; 2019 [updated 2019 cited 2021 Aug 24]. Available from: https://secure.cihi.ca/estore/productSeries.htm?pc=PCC189.

37. Yenipınar A, Koç Ş, Çanga D, Kaya F. Determining Sample Size in Logistic Regression with G-Power. Black Sea Journal of Engineering and Science. 2019;2(1):16-22.

38. Coleman N, Halas G, Peeler W, Casaclang N, Williamson T, Katz A. From patient care to research: a validation study examining the factors contributing to data quality in a primary care electronic medical record database. BMC Family Practice [Internet]. 2015; 16(1):11.

39. Sudeh C-S, Hardeep S, David R, Jill S, Morris R, Aneez E, et al. Missed diagnostic opportunities and English general practice: a study to determine their incidence, confounding and contributing factors and potential impact on patients through

retrospective review of electronic medical records. Implementation Science [Internet]. 2015; 10(1):105.

40. Tremblay J-A, Laramée P, Lamarche Y, Denault A, Beaubien-Souligny W, Frenette A-J, et al. Potential risks in using midodrine for persistent hypotension after cardiac surgery: a comparative cohort study. Annals of intensive care. 2020;10(1):1-8.

41. Kuhns BD, Hannon CP, Makhni EC, Alter J, Mather RC, 3rd, Salata MJ, et al. A Comparison of Clinical Outcomes After Unilateral or Bilateral Hip Arthroscopic Surgery: Age- and Sex-Matched Cohort Study. The American journal of sports medicine. 2017;45(13):3044-51.

42. Ravi B, Pincus D, Wasserstein D, Govindarajan A, Huang A, Austin PC, et al. Association of Overlapping Surgery With Increased Risk for Complications Following Hip Surgery: A Population-Based, Matched Cohort Study. JAMA internal medicine. 2018;178(1):75-83.

43. Oichi T, Chikuda H, Ohya J, Ohtomo R, Morita K, Matsui H, et al. Mortality and morbidity after spinal surgery in patients with Parkinson's disease: a retrospective matched-pair cohort study. Spine journal. 2017;17(4):531-7.

44. Charlson ME, Pompei P, Ales KL, MacKenzie CR. A new method of classifying prognostic comorbidity in longitudinal studies: development and validation. Journal of chronic diseases. 1987;40(5):373-83.

45. Quan H, Li B, Couris CM, Fushimi K, Graham P, Hider P, et al. Updating and validating the Charlson comorbidity index and score for risk adjustment in hospital discharge abstracts using data from 6 countries. American Journal of Epidemiology. 2011;173(6):676-82.

46. Terwee CB, Prinsen C, Chiarotto A, de Vet H, Bouter LM, Alonso J, et al. COSMIN methodology for assessing the content validity of PROMs–user manual. Amsterdam: VU University Medical Center. 2018.

47. Holt TP, Loraas TM. Using qualtrics panels to source external auditors: A replication study. Journal of Information Systems. 2019;33(1):29-41.

48. Streiner DL, Norman GR, Cairney J. Health measurement scales : a practical guide to their development and use. Oxford: Oxford University Press; 2015.

49. Grant JS, Davis LL. Selection and use of content experts for instrument development. Research in nursing & health. 1997;20(3):269-74.

50. O'Brien SMP, Shahian DMMD, DeLong ERP, Normand S-LTP, Edwards FHMD, Ferraris VAMD, et al. Quality Measurement in Adult Cardiac Surgery: Part 2—Statistical

Considerations in Composite Measure Scoring and Provider Rating. The Annals of Thoracic Surgery. 2007;83(4 Supplement):S13-S26.

51. Shahian D, Edwards F, Ferraris V, Haan C, Rich J, Normand S, et al. Quality Measurement in Adult Cardiac Surgery: Part 1—Conceptual Framework and Measure Selection. The Annals of Thoracic Surgery. 2007;83(4 Supplement):S3-S12.

52. Dubois CA, D'Amour D, Tchouaket E, Clarke S, Rivard M, Blais R. Associations of patient safety outcomes with models of nursing care organization at unit level in hospitals. International journal for quality in health care : journal of the International Society for Quality in Health Care. 2013;25(2):110-7.

53. Gearing RE, Mian IA, Barber J, Ickowicz A. A methodology for conducting retrospective chart review research in child and adolescent psychiatry. Journal of the Canadian Academy of Child and Adolescent Psychiatry. 2006;15(3):126-34.

54. Vassar M, Holzmann M. The retrospective chart review: important methodological considerations. Journal of educational evaluation for health professions. 2013;10:12.

55. McHugh ML. Interrater reliability: the kappa statistic. Biochemia medica. 2012;22(3):276-82.

56. Tchouaket E, Boivin S, Robins S, Sia D, Kilpatrick K, Brousseau S, et al. Development and validation of a time and motion guide to assess the costs of prevention and control interventions for nosocomial infections: A Delphi method among experts. PLoS ONE [Internet]. 2020; 15(11).

57. Rym B, Hendy A, Marine L, Olivier S, Corinne A. Using and reporting the Delphi method for selecting healthcare quality indicators: a systematic review. PLoS ONE [Internet]. 2011; 6(6).

58. Polit DF, Beck CT, Owen SV. Is the CVI an acceptable indicator of content validity? Appraisal and recommendations. Research in Nursing & Health. 2007;30(4):459-67.

59. Vaismoradi M, Salsali M, Turunen H, Bondas T. A qualitative study on Iranian nurses' experiences and perspectives on how to provide safe care in clinical practice. Journal of Research in Nursing. 2013;18(4):351-65.

60. Tabachnick, Fidell. Using multivariate statistics. Sixth edition.. ed. Fidell L, editor: Boston : Pearson Education; 2013.

61. Ibrahim JG, Chu H, Chen M-H. Missing data in clinical studies: issues and methods. J Clin Oncol. 2012;30(26):3297.

62. Armstrong RA. When to use the Bonferroni correction. Ophthalmic & physiological optics : the journal of the British College of Ophthalmic Opticians (Optometrists). 2014;34(5):502-8.

63. Daoud JI, editor Multicollinearity and regression analysis. Journal of Physics: Conference Series; 2017: IOP Publishing.

64. Kilpatrick K, Tchouaket E, Carter N, Bryant-Lukosius D, DiCenso A. Structural and Process Factors That Influence Clinical Nurse Specialist Role Implementation. Clinical Nurse Specialist. 2016;30(2):89-100.

65. Olive DJ. Linear regression. Cham, Switzerland: Springer; 2017.

66. Ramakrishnan N, Hanauer D, Keller B. Mining electronic health records. Computer. 2010;43(10):77-81.

figure 1 Click here to access/download;Figure;figure_1.docx

Figure 1. The confounding variables and the level of adherence to best-practice guidelines by interprofessional teams with and without acute care nurse practitioners in cardiac surgery

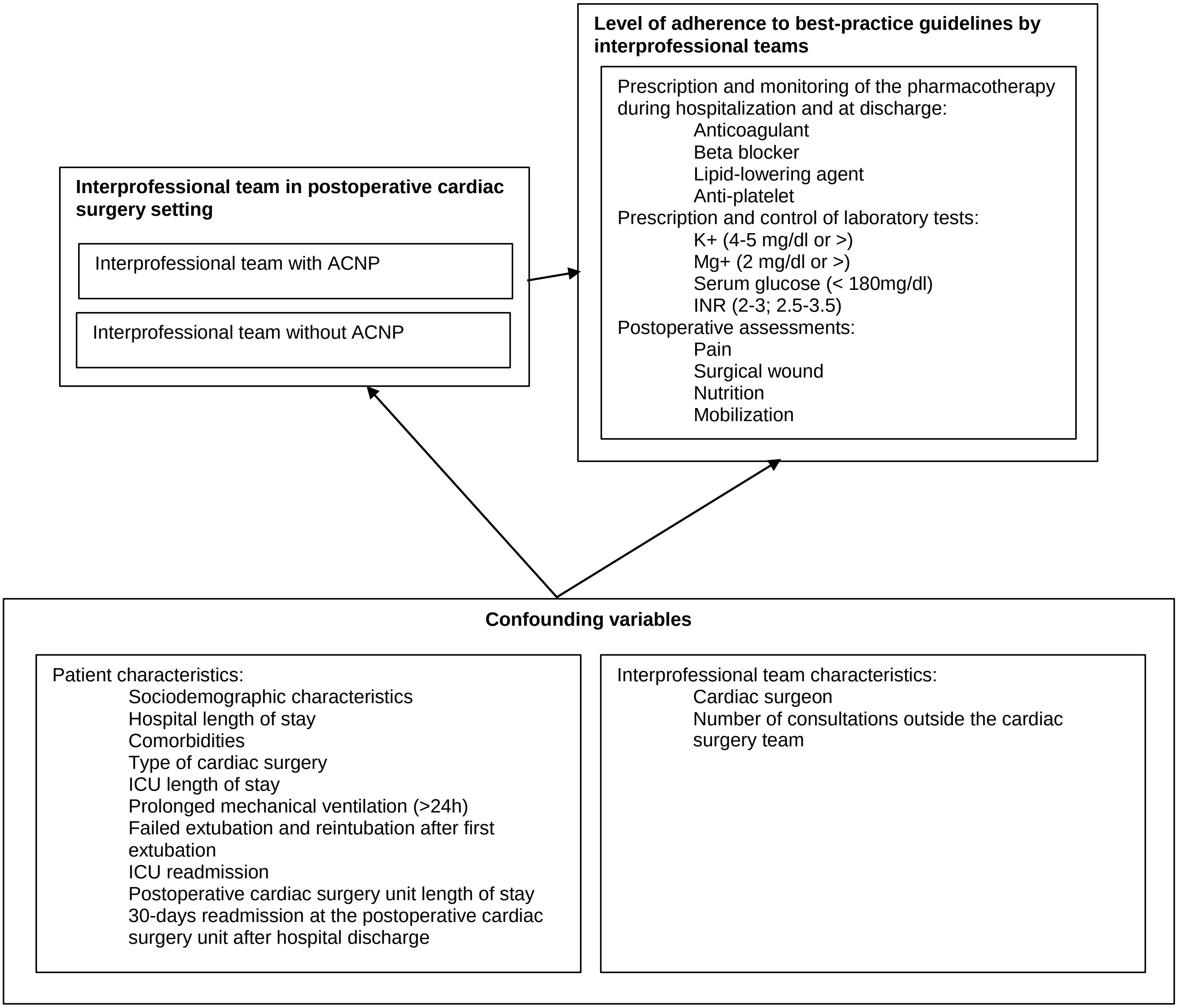

Click here to access/download
Supporting Information
supporting_information.docx

The level of adherence to best-practice guidelines by interprofessional teams with and without acute care nurse practitioners in cardiac surgery: a study protocol

Formatted: Numbering: Continuous

Authors and affiliations: Li-Anne Audet, RN.[1], M.Sc, Mélanie Lavoie-Tremblay, RN.[2], Ph.D, Éric Tchouaket, Ph.D[3], Kelley Kilpatrick, RN., Ph.D[1,4,5]

[1] McGill University, Ingram School of Nursing, Faculty of Medicine and Health Sciences, 680 Sherbrooke West, Suite 1800, Montreal, QC, Canada, H3A2M7

[2] Université de Montréal, Faculté des sciences infirmières, Pavillon Marguerite-d'Youville, 2375 chemin de la Côte-Ste-Catherine, Montreal, QC, Canada, H3T1A8

Formatted: French (Canada)

[3] Université du Québec en Outaouais, Département des sciences infirmières, 5 rue Saint-Joseph, Saint-Jérôme, QC, Canada, J7Z0B7

[4] Centre intégré universitaire de santé et de services sociaux de l'Est-de-l'Île-de-Montréal-Hôpital Maisonneuve-Rosemont (CIUSSS-EMTL-HMR), 5415, boulevard de l'Assomption, Montreal, QC, Canada, H1T 2M4

[5] Susan E. French Chair in Nursing Research and Innovative Practice, McGill University, Ingram School of Nursing, Faculty of Medicine and Health Sciences, 680 Sherbrooke West, Suite 1800, Montreal, QC, Canada, H4A 2M7

Corresponding author: Li-Anne Audet, Ingram School of Nursing, McGill University, 680 Sherbrooke Street West, Montreal, QC, H3A2M7, Email address: li-anne.audet@mail.mcgill.ca

Word count: 5,721

The level of adherence to best-practice guidelines by interprofessional teams with and without acute care nurse practitioners in cardiac surgery: a study protocol

Short title

Adherence to best-practice guidelines by interprofessional teams with and without acute care nurse practitioners

Abstract

Background

Acute care nurse practitioners (ACNP) ~~have been implemented~~ in postoperative cardiac surgery settings ~~and have shown to~~ provide significant benefits to patients and organizations. ~~To explain these associations,~~ recent studies have suggested that ACNP increase the level of adherence to best-practice guidelines by interprofessional teams. However, it is unknown if interprofessional teams with ACNP are associated with higher levels of adherence to best-practice guidelines compared to interprofessional teams without ACNP. Furthermore, no extraction tool is available to measure the level of adherence to best-practice guidelines by interprofessional teams in postoperative cardiac surgery settings. This project aims to examine the level of adherence to best-practice guidelines of interprofessional teams with and without ACNP in a postoperative cardiac surgery setting in Québec, Canada.

Methods

A retrospective observational study will be conducted of 300 patients hospitalized between January 1, 2019 and January 31, 2020 in a postoperative cardiac surgery unit

in Québec, Canada. Data will be collected from patient health records and electronic databases. An extraction tool will be developed based on systematic review of the literature, and will include best-practice guidelines and confounding variables related to patient and interprofessional teams' characteristics. Content and criterion validation, and a pilot-test will be conducted for the development of the tool. A multivariate linear regression model will be developed and adjusted for ~~several~~ confounding variables, in order to examine the association between interprofessional teams with and without ACNP, and ~~the~~ level of adherence to best-practice guidelines.

Discussion

This project represents the first study to examine the level of adherence to best-practice guidelines by interprofessional teams with and without ACNP in a postoperative cardiac surgery setting. The findings of this project will generate empirical data focusing on the contribution of ACNP within interprofessional teams, and ultimately enhance the delivery of high quality and evidence-based care for patients and families.

Keywords

Acute care nurse practitioner, best-practice guidelines, cardiac surgery, interprofessional teams, quality of care

Background

Cardiac surgeries are one of the leading types of surgical procedures performed internationally. In 2017, approximately 1 million patients throughout the world underwent a cardiac surgery (1). Most ~~of~~ cardiac surgeries are performed in developed countries (1). In 2018, the Society of Thoracic Surgeons (STS) reported 287,872 cardiac surgeries and procedures performed in the United States (2). In Canada, 42,989 coronary artery bypass grafts (CABG) and 7,186 valve repairs were performed between 2013 and 2016 (3, 4).

In the postoperative phase after ~~the~~ surgery, many patients are at high risk of developing adverse events and postoperative complications such as myocardial infarction and wound infection (5). These complications are associated with higher risk of mortality and development of comorbidities (e.g., heart failure), as well as a decrease in the well-being and quality of life of patients and families (6). For healthcare organizations, adverse events and postoperative complications are associated with a longer length of stay at the hospital, higher rates of readmission in intensive care units, higher rates of surgical re exploration, and an increase in cost (7, 8).

To prevent the risk of adverse events and postoperative complications for patients and families, international healthcare organizations and research teams have developed best-practice guidelines (7, 9). Best-practice guidelines are defined as evidence-based practice of care, and aim to ensure the performance of interprofessional teams, enhancing the quality and safety of the care given to patients and families. In postoperative cardiac surgery settings, best-practice guidelines related to the pharmacotherapy, laboratory tests, clinical indicators, and lifestyle promotion

Formatted: Highlight

(e.g., diet, cardiac rehabilitation) have been developed and implemented in healthcare centres (2).

A consensus in the literature supports the importance of a high level of adherence by interprofessional teams to best-practice guidelines, in order to ensure their efficacy (7, 10). Adherence to best-practice guidelines is defined as the achievement of the guideline, as well as the associated interventions performed by clinicians (11). In surgical settings (e.g., neurosurgery, orthopedic surgery) international research teams (12-15) have developed composite scores to examine the level of adherence to best-practice guidelines by interprofessional teams. Their findings suggested that a higher level of adherence is associated with a higher quality of care given to patients and families.

In postoperative cardiac surgery settings, recent studies (5, 16) identified a significant association between a high level of adherence to best-practice guidelines by interprofessional teams and a lower risk of postoperative complications and adverse events for patients. Other studies (5, 16, 17) have shown a significant association between a lower level of adherence to best-practice guidelines by interprofessional teams and a higher risk of postoperative complications and adverse events for patients. Larrazzet et al. (2014) conducted a longitudinal study of 144 patients who died after cardiac surgery. The authors identified a significant association between a lack of adherence to best-practice guidelines by interprofessional teams, and a higher risk of mortality, highlighting the importance of the high adherence to best-practice guidelines by interprofessional teams (17).

Internationally, there is variability in the scope of practice and level of autonomy of ACNPs (18,19). Recent studies have examined the practice of nurse practitioners (NP) within primary and acute care settings, and suggested that their practice could increase the level of adherence to best-practice guidelines by interprofessional teams (18, 19). The NP’s support of the practice of different care providers in the team with a focus on best evidence is believed to underpin providers’ adherence to best-practice guidelines focused on pharmacotherapy, clinical indicators, and lifestyle promotion. NPs also enhance collaboration and communication among teams’ members and facilitate continuity of care, contributing to the higher adherence to best-practice guidelines by interprofessional teams (18, 20).

Based on the International Council of Nurses guidelines, the NP is an advanced practice nursing role, based on a graduate or postgraduate education and an in-depth nursing and medical expertise (21-23). In several acute care settings, including postoperative cardiac surgery, acute care nurse practitioners (ACNP) have been implemented in interprofessional teams and performed activities such as the clinical and psychosocial assessment of patients, the prescription and management of pharmacotherapy, laboratory tests and clinical interventions, lifestyle promotion, teaching of coping strategies, preparation for hospital discharge, and the management of consultations and external resources (24). International research teams (23, 25) have examined the practice of ACNPs within postoperative cardiac surgery settings. They identified significant associations between the ACNPs’ practice and higher patient satisfaction, higher participation of patients to cardiac rehabilitation, and lower length of stay at the hospital after the cardiac surgery. However, other research teams (21, 26)

Formatted: Highlight

Formatted: Highlight

Formatted: Highlight

did not identify significant associations between ACNP practice and patient and organizational outcomes within postoperative cardiac surgery settings. More specifically, the authors suggested that the difficulty of to recruiting participants, the high attrition rate, the small sample sizes, and the inability to adjust the data analysis for confounding variables related to the patient and interprofessional teamsteams' characteristics were important limits contributing to the non-significant findings (21,26).

Formatted: Highlight

Systematic reviews of randomized controlled trials (RCT) (21, 23) have been conducted to understand the inconsistent findings in the current literature. These systematic reviews have identified three important limits contributing to the inconsistent findings of the current literature. Firstly, although several RCT of the current literature focus on the efficacy of ACNP for patients and healthcare organizations, less attention has been paid to the efficacy of these providers for interprofessional teams. The qualitative study of Reich et al. (2018) suggested that the implementation of ACNP within interprofessional teams increases the level of adherence of those teams to best-practice guidelines in postoperative cardiac surgery settings, and represents an underlying factor contributing to the efficacy of NP (19). However, these propositions have not been subjected to statistical validation, thus it is unknown if interprofessional teams with ACNP are associated with higher levels of adherence to best-practice guidelines, compared to interprofessional teams without ACNP. Moreover, until today, no validated extraction tool is available to measure the level of adherence to best-practice guidelines of interprofessional teams with and without ACNP in postoperative cardiac surgery settings.

Secondly, systematics reviews have highlighted the difficulty in adequality controlling confounding variables in the existing RCT (21, 23). Confounding variables represent important factors influencing the efficacy of ACNP on patient and organizational outcomes in postoperative cardiac surgery settings. Methodological limits of the existing RCT, such as the small sample size and high attrition rate, limit the capacity of research teams to develop robust multivariate statistical models adjusted for confounding variables related to patient, interprofessional teams, and organizational characteristics (21, 23). Interestingly, retrospective observational studies have shown to be a relevant alternative to examine the association between ACNP and patient and organizational outcomes in postoperative cardiac surgery settings. Existing studies (27-30) have used retrospective observational studies to gather a large cohort of patients, limit attrition, and develop statistical models adjusted for many confounding variables. These studies (6, 27-29) have identified significant associations between ACNP within interprofessional teams, and lower risks of mortality and decreased costs in postoperative cardiac surgery settings.

Thirdly, the majority of the RCT focus on ACNP practice within primary care settings (e.g., in-home care, rehabilitation clinic) after the cardiac surgery. Less attention has been paid to ACNP practice in acute care settings; however, within these settings, ACNP works in collaboration with cardiac surgeons, nursing teams, rehabilitation teams, social workers, and other members of the interprofessional teams to ensure the patient optimal recovery after the surgery, and prevents the risk of adverse events and postoperative complications. Moreover, for patients and families, the acute care hospitalization after the surgery represents a crucial phase of their

Formatted: Highlight

Formatted: Highlight

Formatted: Highlight

postoperative recovery ~~and is~~ associated with several physical, psychosocial, and emotional stressors (30). Future studies are needed to substantiate the contribution of ACNP in interprofessional teams within acute care settings, and identify the benefits of their practice for patients, families, interprofessional teams, and healthcare organizations.

~~Consequently,~~ the aim of this project is to examine the level of adherence to best-practice guidelines of interprofessional teams with and without ACNP in acute postoperative cardiac surgery settings in Québec, Canada.

Study hypothesis and objectives

The hypothesis of this study is:

H1: This study will verify if interprofessional teams with ACNP are associated with a higher level of adherence to best-practice guidelines compared to interprofessional teams without ACNP, after controlling for the patient and interprofessional team characteristics.

The objectives of this study are as follows:

1. Develop and pilot-test an extraction tool to measure ~~the~~ level of adherence to best-practice guidelines of interprofessional teams within postoperative cardiac surgery setting.
2. Describe the patient and interprofessional teams' characteristics, as well as the level of adherence to best-practice guidelines, of patients under the care of interprofessional teams with and without ACNP.
3. Examine the association between interprofessional teams with ACNP and the level of adherence to best-practice guidelines, compared to interprofessional

Formatted: Highlight

Formatted: Highlight

Formatted: Highlight

teams without ACNP, after adjusting patient and interprofessional team characteristics in postoperative cardiac surgery setting.

Methods and design

Study design

A retrospective observational study (31, 32) will be conducted. De-identified data will be extracted from the UHC data warehouse, and the patient health records to assemble the retrospective cohort of patients and conduct the data collection. Ethics approval was obtained from the McGill University Health Centre Research Ethics Board on September 15, 2021 (IPSSA chirurgie cardiaque/2022-8094). This project uses the guidelines from *Strengthening the Reporting of Observational Studies in Epidemiology (STROBE) Statement: Guidelines for Reporting Observational Studies* (32) were used for this project (SA appendix).

Formatted: Font color: Red, Highlight

Study setting

Formatted: Highlight

This study will be conducted at a University healthcare centre (UHC) in Québec, Canada. Annually, this healthcare centre performs approximately 1,000 cardiac surgeries and has a 36-bed postoperative cardiac surgery unit.

After cardiac surgery, patients are admitted to the intensive care unit (ICU), and are under the care of ICU teams. At the time of data collection, the ICU teams in Québec, Canada do not include ACNP. On average, patients are hospitalized for 24 hours in the ICU before being transferred to the postoperative cardiac surgery unit. Patients readmitted to the ICU are under the care of ICU teams.

Upon admission to the postoperative cardiac surgery unit, patients are assigned to the care of interprofessional teams with or without ACNP. Team assignment depends

on the current workload of each team, and the availability of the beds in the unit. Interprofessional teams with ACNPs are assigned 16 of the unit's beds (44%) and interprofessional teams without ACNP are assigned 20 of the unit's beds (56%). Patients are followed by their respective teams from their admission to the cardiac surgery unit, until the hospital discharge or death. On average, the length of stay at the hospital after the surgery ranges from 9 to 11 days (33).

Interprofessional teams with and without ACNP include the following: cardiac surgeons and residents nursing teams, skin care and wound therapists, physiotherapists, nutritionists, respiratory therapists, social workers, and medical and other specialists. Interprofessional teams with and without ACNP ensure the daily follow-up of patients, the assessment of the patient's clinical and psychosocial condition, the management of pharmacotherapy, clinical intervention, laboratory tests, lifestyle promotion, and the preparation of patients upon hospital discharge.

The practice of acute care nurse practitioners in postoperative cardiac surgery unit

Since 2017, seven ACNPs practice in postoperative cardiac surgery, based on a monthly rotation system. Two ACNP simultaneously practice within interprofessional teams and collaborate with other clinicians to ensure the daily follow-up of the patients after cardiac surgery. ACNP practice includes the assessment of the clinical and psychosocial condition of the patient and families, the management of ~~the~~ pharmacotherapy, clinical interventions, and laboratory tests. The ACNP also teaches coping strategies, lifestyle promotion, cardiac rehabilitation, preparation of the patients and families for the in-home recovery after discharge. The ACNP supports the

Formatted: Highlight

preparation for -hospital discharge (e.g., prescription of the patient medication after discharge, -summary for the family physician, etc.) (34).

Study population and sample

A ~~dynamic~~ cohort of patients will be assembled from hospitalized patients at the postoperative cardiac surgery unit between January 1, 2019, and January 31, 2020. This timeframe will avoid the recruitment of patients during the COVID-19 pandemic, a window of time during which the practice of ACNP within the unit was inconsistent. Patients will be selected, based on three inclusion criteria: 1) patient admitted for a diagnosis of CABG and/or valve repair, 2) patient age is over 18 years old, and 3) patient has been hospitalized for at least 24 hours in the postoperative cardiac surgery unit. The minimum 24-hour criteria will allow our team to examine the hospitalization of the patient in the postoperative cardiac surgery unit, which is the setting where ACNPs practice in Québec (35). Patients will be identified from the electronic databases of the UHC and the diagnostic codes of the Canadian Institute for Health Information (36) (SB appendix).

A sample size calculation was conducted in G*power (37). This project will include 15 variables, including one independent variable, one dependent variable, and 13 confounding variables. The variables selected for this project are described in the next section. To perform one multivariate linear regression model and detect an effect size of f^2=0.15 with a power of 0.80 and a standard error of 5%, a minimum sample size of 183 patients was estimated.

A total of 300 patients will be selected, based on the sample size calculation and an over-estimation of at least 20%, to consider the risk of error from the presence of

Formatted: Highlight

missing data and incomplete patient health records (38, 39). A systematic random selection of 150 patients under the care of interprofessional teams with ACNP will be conducted and matched with 150 patients under the care of interprofessional teams without ACNP. Three criteria (40-43) will be used to match each pair (1:1): 1) age (i.e., a five-year gap will be tolerated) 2) sex, and 3) type of cardiac surgery (i.e., CABG, valve repair, or CABG/valve repair).

Patients will be followed from their admission to the ICU after the cardiac surgery, until the occurrence of the following events, whichever occurs first: 1) discharge from the hospital, 2) death, or 3) the cumulation of 14-days in hospital after the surgery. The 14-days' timeframe will allow our team to capture the acute phase of the patient hospitalization after the cardiac surgery (33). The total length of stay for patients hospitalized longer than 14-days will be measured.

Variables under study

A systematic review of RCTs (24) and an extensive search in the literature and international healthcare organizations (e.g., American Heart Association, Society of Thoracic Surgeons) was conducted to retrieve best-practice guidelines for interprofessional teams in postoperative cardiac surgery settings, and confounding variables related to patient and interprofessional teams' characteristics. A total of 12 best-practice guidelines and 13 confounding variables were identified and presented in Figure 1. The operationalization of each variable is presented in SC appendix.

Independent variable: interprofessional teams with and without acute care nurse practitioner

A dichotomous variable will be created to measure the inclusion of at least one ACNP within the interprofessional team. For patients under the care of an interprofessional team with ACNP, an additional descriptive variable will measure the number of days where the ACNP was implicated in the daily follow-up of the patient, on the entire hospitalization at the postoperative cardiac surgery unit.

Dependant variable: the level of adherence to best-practice ~~guidelines~~ by interprofessional teams

A composite score will be developed to measure the level of adherence to best-practice ~~guidelines~~ by interprofessional teams. An individual composite score will be calculated per patient per day of hospitalization at the postoperative cardiac surgery unit. A total of 12 best-practice guidelines will be included in the composite score (Figure 1). Each best-practice guideline will be scored on two points based on the following: 1) the achievement of the guideline recommendation (e.g., prescription of beta blocker) and 2) the associated interventions performed by clinicians (e.g., follow-up of the blood pressure). All best-practice guidelines are divided into three categories: 1) pharmacotherapy (n=4), 2) laboratory tests (n=4), and 3) postoperative assessment (n=4).

Four best-practice guidelines are included in the category of pharmacotherapy. These guidelines are the prescription and monitoring of medications including: 1) anticoagulants, 2) beta blockers, 3) lipid-lowering agents, and 4) anti-platelets, during hospitalization and at hospital discharge of the patient. For each best-practice guideline, two additional descriptive variables will be collected, including the type of medication

prescribed and the presence of a contraindication, which precludes the possibility of achieving the guideline recommendation (e.g., allergy).

Four best-practice guidelines on laboratory tests are included, recommending the prescription and monitoring of the following: 1) potassium (K+), 2) magnesium (Mg+), 3) serum glucose, and 4) international normalized ratio (INR) by interprofessional teams during the patient's hospitalization in the postoperative cardiac surgery unit. The achievement of each best-practice guideline recommendation will be confirmed if the laboratory result falls under the normal range supported by the current literature. An additional variable called "no INR needed" will be added specifically for the best-practice guideline related to the prescription and management of INR, to take into consideration patient who don't need a strict INR follow-up (e.g., administration of direct acting oral anticoagulant (DAOC)). A pilot test will be performed to assess the characteristics of the patients hospitalized at the post-operative cardiac surgery unit and the feasibility of the data collection. The following three additional descriptive variables will be measured for each guideline: 1) number of prescriptions requested by interprofessional teams, 2) proportion of abnormal values below or above normal range, and 3) average of all values of laboratory tests.

Formatted: Highlight

Four best-practice guidelines are included in the category of the postoperative assessment performed by interprofessional teams. A daily assessment of the patient's level of pain, surgical wound(s), nutrition, and mobilization will be measured. For each best-practice guideline recommendation, the achievement of the guideline will be confirmed if at least one postoperative assessment per day is performed by the ACNP or other members of the interprofessional teams during the patient's hospitalization in

the postoperative cardiac surgery unit. Two additional descriptive variables will be measured for each best-practice guideline, including: 1) the total number of postoperative assessments performed by interprofessional teams during the hospitalization, and 2) the type of associated interventions conducted by clinicians (e.g., non-pharmacological, consultation, etc.).

Confounding variables related to patient characteristics

Eleven confounding variables related to patient characteristics will be measured. For each patient, sociodemographic characteristics (e.g., sex, age) will be measured. The total length of stay at the hospital will be measured, which includes the stay in the ICU and postoperative cardiac surgery unit. The type of cardiac surgery will be collected from a categorical variable with three categories: 1) CABG, 2) valve repair, and 3) CABG/valve repairs. Comorbidities will be measured and operationalized from the Charlson Comorbidity Index (CCI) (44, 45). A total score of 24 points will be calculated for each patient based on the 17 clinical conditions included in the CCI (SD appendix). The length of stay in the postoperative cardiac surgery unit will be collected for each patient. The hospital 30-days readmission at the postoperative cardiac surgery unit after discharge will be measured for each patient from a dichotomous variable.

Four confounding variables related to the patient characteristics in the ICU setting will be measured. The total number of hours hospitalized in the ICU will be measured from the time (in hours) of admission to the ICU after the surgery and the time (in hours) of discharge of the patient. The postoperative prolonged mechanical ventilation will be measured from a dichotomous variable. A prolonged mechanical ventilation will be confirmed if the time under mechanical ventilation exceeds 24 hours.

The failed extubation of the patient and reintubation after first extubation will be measured with a dichotomous variable. The number of episodes of ICU readmission after initial discharge will be measured for each patient.

Confounding variables related to interprofessional teams' characteristics

Two confounding variables related to interprofessional teams' characteristics will be measured. First, the cardiac surgeon who performed the surgery and ensured the follow-up of the patient's recovery will be noted. A confidential code will be created for each surgeon practising in the UHC. Second, a discrete variable will be created to measure the number of consultations conducted by clinicians from different expertises (e.g., nutrition, rehabilitation team) during the patient's hospitalization in the postoperative cardiac surgery unit. A categorical descriptive variable will be created to measure each clinician's expertise, including the following: 1) rehabilitation team (e.g., physical therapist, occupational therapist), 2) respiratory therapist, 3) social worker, 4) skin and wound care therapist, 5) nutritionist, and 6) speciality consultation (e.g., internal medicine, nephrology).

Data collection

An extraction tool will be developed and pilot-tested, based on the best-practice guidelines and the confounding variables related to the patient and interprofessional team characteristics (Figure 1). One extraction will be completed per patient per day during the hospitalization in the postoperative cardiac surgery unit, by the first author or a research assistant. The development of the extraction tool followed the Consensus-based Standards for the selection of health measurement instrument (COSMIN)

Formatted: Highlight

guidelines (46) and will include three stages: the content validation; the criteria validation; and the pilot-test.

Data collection for the content validation

Content validation will be performed following two steps (46). First, an expert committee will be recruited from clinicians, managers, and researchers who have expertise in postoperative cardiac surgery. The identification and recruitment of experts will be conducted by our research team and will include experts in Canada. A total of 10 experts will be recruited, including at least the following five: 1) an ACNP who worked a minimum of one year within a postoperative cardiac surgery unit outside of our associated UHC, 2) a cardiac surgeon or fellow in cardiac surgery who worked at least two years in an acute care centre outside of our associated UHC, 3) a nurse manager who manages, at least one year, a postoperative cardiac surgery unit, within our associated UHC, 4) a healthcare professional who worked in the data warehouse of our associated UHC for at least one year, and 5) a researcher in nursing or healthcare sciences with an expertise in measurement instruments and/or cardiac surgery.

Second, an electronic survey (47) will be created and composed of all items of the extraction tool (best-practice guidelines and confounding variables). For each item, experts will assess its relevance using a 5-points Likert scale (48, 49). Additional spaces for qualitative comments and suggestions will be included in the survey. Two rounds of revision are planned. After the first round, our team will update the extraction tool and revise any unclear items based on the experts' recommendations, and an updated version will be resubmitted to the expert committee.

Data collection for the criterion validation

The criterion validation of the extraction tool will follow three consecutive steps. Firstly, the best-practice guidelines of The Society of Thoracic Surgeons (STS) have been selected as the gold standard of comparison for this study. For many years, the STS has been a leading healthcare organization in the development and validation of best-practice guidelines and performance measures in the care of patients in cardiac surgery settings (10). In 2007, the STS developed and validated 21 performance measures for the delivery of high quality of care for patients who underwent CABG, valve repair, and CABG/valve repairs (50, 51). For this project, five performance measures were selected, including: 1) prolonged mechanical ventilation higher than 24 hours in the ICU; 2) a 30-day readmission rate at the postoperative cardiac surgery unit after hospital discharge; and prescription of the 3) anti-platelet, 4) beta blocker, 5) and lipid-lowering agent, during the hospitalization at the postoperative cardiac surgery unit and at discharge. Each performance measure is operationalized as the proportion of patients who achieved the outcome (e.g., proportion of patients who were under prolonged mechanical ventilation) over the total sample size (46, 47).

Secondly, 30 patient health records will be reviewed independently by two reviewers on two occasions, including the first author and a research assistant. For the first round of revision, the patient's health record will be reviewed with the extraction tool. For the second round of revision, the records will be reviewed with the five performance measures from the STS.

Formatted: Highlight

Data collection for the pilot-test

A pilot-test will be conducted to assess the feasibility of the tool, standardize data collection among reviewers, examine the quality and accessibility of the retrospective

data, and update the tool if needed. Four iterative steps will be included in the pilot-test (52-55). First, two reviewers will conduct the pilot-test and data collection. Training sessions and documentation will be given to the reviewers.

Secondly, 30 patient health records from hospitalized patients in the postoperative cardiac surgery unit between January 1, 2019, and January 31, 2020, will be randomly selected. These patients will not be included in the retrospective cohort. Data will be collected by the two reviewers with the extraction tool. Inter-rater reliability will be assessed and a Cohen kappa higher than 0.60 will be targeted (55).

Thirdly, a triangulation of the available data will be performed to assess the quality and accessibility of the data. Multiple data sources will be screened between the electronic databases and patient health records. Items from the extraction tool with a high quantity of missing data or low quality of available data will be modified or removed.

Finally, frequent meetings among all research team members will be conducted to discuss divergences, reinforce convergences, and establish a consensus. The extraction tool will be updated after the pilot-test if needed (52).

During data collection from the retrospective cohort of patients, an additional 30 patient health records will be reviewed by two independent reviewers to ensure the standardization of the data collection (49, 50). Inter-rater reliability will be assessed and a Cohen Kappa value higher than 0.60 will be targeted (55).

Data analysis

Statistical analysis will be conducted to meet objectives one, two and three. The unit of observation will be the patient-level and the unit of analysis will be the interprofessional team-level.

Objective 1

The content validation of the extraction tool will include two rounds of revisions, with additional rounds if needed (56, 57). For each round of revision, a Fleiss Kappa will be calculated and a value between 0.60 and 0.80 will be targeted (48, 49). The content validity index (CVI) of each individual item of the extraction tool will be calculated from the proportion of experts who rated a score of 4 or 5 on the Likert scale (58). A CVI of 0.80 will be targeted for each item and items with a value below 0.80 will be removed or modified. Then, the proportion of items with a CVI value above 0.80 will be calculated to determine the overall CVI of the extraction tool. A value higher than 0.80 for the overall CVI of the extraction tool will be targeted (46). Qualitative comments and recommendations by experts will be analyzed by content analysis (59).

The criteria validation will be assessed using 30 patient health records. Five performance measures developed by the STS will serve as the gold standard for the comparison (10). First, the proportion of patients who achieved each performance measure over the total sample size (n=30) will be calculated, from the measures collected with the gold standard, as well as the measures collected with the extraction tool. Second, Spearman correlation will be calculated to assess the correlation between the proportions obtained from the measures of the gold standard, and the proportions obtained from the measures of the extraction tool. Values of Spearman correlation higher than 0.70 will be targeted (48, 49).

Objective 2

Descriptive analysis will be performed to assess the level of adherence by interprofessional teams with and without ACNP, as well as the confounding variables for patients under the care of both teams (60). An analysis of missing data will be conducted to examine the quality and distribution of missing data within patient health records and electronic databases. Team meetings and consultations with clinicians and statisticians will be held to discuss and identify potential causes of missing data, as well as to conduct a statistical analysis to manage them (61).

A composite score will be developed to measure the level of adherence to best-practice guidelines by interprofessional teams with and without ACNP. A composite score per patient per day of hospitalization in postoperative cardiac surgery will be developed and based on four consecutive steps. Firstly, for each best-practice guideline, a score on a scale of two will be created and points will be attributed for: 1) the achievement of the best-practice guideline recommendation, and 2) the associated intervention performed by clinicians. Points will be given based on the information contained in the patient health record and electronic databases. A missed intervention will be considered as a lack of adherence to best-practice guidelines, and no point will be given.

Secondly, all best-practice guidelines are divided into three categories: 1) pharmacotherapy, 2) laboratory tests, and 3) postoperative assessments. The sum of the individual score of each best-practice guideline will be calculated for each category. More specifically, each category includes four best-practice guidelines, and will be based on a total score ranging from zero to eight.

Thirdly, an overall composite score will be calculated from the sum of all the scores of the three categories, ranging from zero to 24. The overall composite score will be transformed into a value in percentage (0 to 100%) and an increase of the score will be associated with an increase of the level of adherence to best-practice guidelines by interprofessional teams. An overall score will be calculated per patient per each day hospitalized in the postoperative cardiac surgery unit.

Finally, an average composite score will be calculated from the average of all overall scores during the patient's hospitalization at the postoperative cardiac surgery unit. An average composite score will be calculated for all patients included in the retrospective cohort. A graphic illustration will be created from all average scores to visualize the tendency and distribution of the scores of the cohort.

Objective 3

Bivariate analysis will be conducted, and the statistical significance will be based on a *p*-value of 0.05 (60). The Bonferroni correction will be applied for the examination of each confounding variable related to the patient and interprofessional teams' characteristics (62).

A multivariate linear regression model will be developed to examine the association between interprofessional teams with and without ACNP and the level of adherence to best-practice guidelines (63-65). An examination of the distribution of the data will be performed. In the case of an abnormal distribution of the data, a transformation will be conducted to adjust the statistical model based on the observed data. The independent variables will include the interprofessional teams with and without ACNP, as well confounding variables. A correlation matrix will be developed,

and tolerance value lower than 0.2 and a variance inflation factor (VIF) value higher than 5 will be used to identify multicollinearity between independent variables (63-65). Independent variables with multicollinearity will be removed one at the time and a new regression will be calculated. The dependent variable of the regression model will be the average composite score (%) of the level of adherence to best-practice guidelines. Statistical significance will be based on a *p*-value of 0.05. Sensitivity analysis will be conducted based on a selection of confounding variables related to the characteristics of the patient (e.g., length of stay, ICU readmission). The selection of these confounding variables will be based on the observed data and descriptive analysis. The residual analysis and the analysis of the R^2 will be conducted to assess the goodness-of-fit of the multivariate linear regression model.

Formatted: Highlight

Interpretation of the findings

Consultations with clinicians and managers practising in postoperative cardiac surgery settings in Québec and Canada will be conducted to gain an in-depth understanding of the findings. These consultations will allow our team to explore potential underlying factors and mechanisms which could influence the level of adherence to best-practice guidelines by interprofessional teams with and without ACNP. Field notes will be taken during these consultations and analyzed using content analysis (45).

Ethical considerations

Ethics approval was obtained prior to the beginning of the study. No identifying information will be collected during the content validation with the expert committee and the data collection within electronic databases and patient health records. A confidential pairing system will be created to match the patient identification with a random number

(66). The database of the project will be kept in a secure server, protected by a confidential numeric code known only by the research team. During the dissemination of the findings, data will be shared in an aggregated form and the identity of the UHC, as well as the identity of the patients and clinicians, will remain confidential.

Discussion

ACNP~~s~~ hold an important place within interprofessional teams caring for patients and families following cardiac surgery. For patients and families, ACNP~~s~~ contribute to the management of pharmacotherapy, clinical and psychosocial conditions, as well as lifestyle promotion and teaching of coping strategies. For interprofessional teams, ACNPs enhance the collaboration and communication among team members and reinforce continuity of care. Until now studies examined the efficacy of ACNP on patient and organization outcomes within postoperative cardiac surgery settings; however, less attention has been paid to underlying factors contributing to the effect of ACNPs on these outcomes.

The findings of this study will further our understanding of the contributions of ACNPs in interprofessional teams~~,~~ practising in postoperative cardiac surgery settings in Québec, Canada ~~and internationally~~. This project will generate empirical data to support and document the practice of ACNP~~s~~ within interprofessional teams in four important ways. Firstly, as part of this project, a systematic review of randomized controlled trials was published to retrieve the current literature on~~f~~ the efficacy of ACNP and advanced practice nursing roles in postoperative cardiac surgery settings, and to examine their adherence to best-practice guidelines (40). Secondly, an extraction tool focusing on the level of adherence to best-practice guidelines by interprofessional

Formatted: Highlight

teams in postoperative cardiac surgery will be available for the field of cardiac surgery research~~,~~ and the scientific community more broadly. Thirdly, for patients under the care of interprofessional teams with and without ACNP, the findings will provide a description of the patient and interprofessional team characteristics~~,~~ ~~and~~as well as ~~the~~ adherence to best-practice guidelines ~~for patients under the care of interprofessional teams with and without ACNP~~. Finally, this study will examine the effect of ACNP~~s~~ on ~~the~~ adherence to best-practice guidelines in settings where ACNPs are incorporated within inter-professional teams, and provide ~~evidence~~empirical data for those settings looking to introduce ~~-~~ such roles ~~,~~ in a postoperative cardiac surgery setting.

Funding

This project is funded by the Réseau de recherche en intervention en sciences infirmières du Québec (RRISIQ). Li-Anne Audet holds doctoral scholarships from the Fonds de recherche du Québec – santé as well as from the Québec Ministry of Education (Ministère de l'Éducation et de l'Enseignement Supérieur du Québec). Éric Tchouaket holds a career award from the Fonds de recherche du Québec – Santé (Junior 2 Research Salary Award). Kelley Kilpatrick holds a career award from the Fonds de recherche du Québec – Santé (Senior Research Salary Award) and holder of the Susan E. French Chair in Nursing Research and Innovative Practice. No funding sources were involved in the study conception and design, data collection, analysis, interpretation, or in the final decision to submit this manuscript for publication.

Competing interest

None to declare.

Acknowledgments

Formatted: Highlight

Formatted: Highlight

The authors would like to acknowledge the comments received from Christine Maheu, Andraea Van Hulst, and Alain Biron.

Author Contributions

Conceptualization: Li-Anne Audet, Mélanie Lavoie-Tremblay, Éric Tchouaket, Kelley Kilpatrick

Methodology: Li-Anne Audet, Mélanie Lavoie-Tremblay, Éric Tchouaket, Kelley Kilpatrick

Supervision: Mélanie Lavoie-Tremblay, Éric Tchouaket, Kelley Kilpatrick

Writing – original draft: Li-Anne Audet

Writing – review & editing: Mélanie Lavoie-Tremblay, Éric Tchouaket, Kelley Kilpatrick

References

1. Caplan LR. Primer on cerebrovascular diseases. London, United Kingdom: Academic Press is an imprint of Elsevier; 2017.

2. Bowdish ME, D'Agostino RS, Thourani VH, Desai N, Shahian DM, Fernandez FG, et al. The Society of Thoracic Surgeons Adult Cardiac Surgery Database: 2020 Update on Outcomes and Research. The Annals of thoracic surgery. 2020;109(6):1646-55.

3. Philippon F, Racine N, Labrecque N, Paradis M-J. Réseau québécois de cardiologie tertiaire. Rapport d’activité 2010-2013. Québec, QC; 2013.

4. Canadian Institute of Health Information. Cardiac Care Quality Indicators Report. Ottawa, ON: Canadian Institute for Health Information
; 2017.

5. Bourgon Labelle J, Audet L, Farand P, Rochefort C. Are hospital nurse staffing practices associated with postoperative cardiac events and death? A systematic review. PLOS ONE [Internet]. 2019; 14(10).

6. Lane-Fall MB, Ramaswamy TS, Brown SES, He X, Gutsche JT, Fleisher LA, et al. Structural, Nursing, and Physician Characteristics and 30-Day Mortality for Patients Undergoing Cardiac Surgery in Pennsylvania. Critical care medicine. 2017;45(9):1472-80.

Formatted: French (Canada)

Formatted: French (Canada)

7. Benjamin JE, Virani SS, Callaway WC, Chamberlain MA, Chang RA, Cheng ES, et al. Heart Disease and Stroke Statistics—2018 Update: A Report From the American Heart Association. Circulation. 2019;137(12):e67-e492.

8. Goldfarb M, Bendayan M, Rudski LG, Morin JF, Langlois Y, Ma F, et al. Cost of Cardiac Surgery in Frail Compared With Nonfrail Older Adults. The Canadian journal of cardiology. 2017;33(8):1020-6.

9. McConnell G, Woltz P, Bradford WT, Ledford JE, Williams JB. Enhanced recovery after cardiac surgery program to improve patient outcomes. Nursing. 2018;48(11):24-31.

10. The Society of Thoracic Surgeons. Performance Measures 2019 [internet],Chicago (USA):The Society of Thoracic Surgeons; 2019 [updated 2019; cited 2021 Aug 24]. Available from: https://www.sts.org/quality-safety/performance-measures].

11. Khalil V, Danninger M, Wang W, Khalil H. An audit of adherence to heart failure guidelines in an Australian hospital: A pharmacist perspective. Journal of Evaluation in Clinical Practice. 2017;23(6):1195-202.

12. Burnier M, Egan BM. Adherence in Hypertension: A Review of Prevalence, Risk Factors, Impact, and Management. Circulation Research. 2019;124(7):1124-40.

13. Chan W, Chen A, Tiao D, Selinger C, Leong R. Medication adherence in inflammatory bowel disease. Intestinal Research [Internet]. 2017; 15(4):434-45.

14. López-Campos JL, Quintana Gallego E, Carrasco Hernández L. Status of and strategies for improving adherence to COPD treatment. International Journal of COPD [Internet]. 2019; 14:1503-15.

15. Moon SJ, Lee WY, Hwang JS, Hong YP, Morisky DE. Accuracy of a screening tool for medication adherence: A systematic review and meta-analysis of the Morisky Medication Adherence Scale-8. PloS one. 2017;12(11):e0187139.

16. Witwer M, Dobbins G, Uher C, McFarren M. Auditing CAUTI Best Practices Bundle Adherence in a 24-bed Neurovascular/Cardiovascular/Intensive Care Unit. AJIC: American Journal of Infection Control. 2019;47(6 Supplement):S30.

17. Larrazet F, Folliguet T, Laborde F, Bachet J, Hervé C. Mortality meetings: ethical considerations and adherence to evidence-based practice from 6 years of experience in a mixed cardiac department. European journal of cardio-thoracic surgery : official journal of the European Association for Cardio-thoracic Surgery. 2011;40(5):1039-45.

Formatted: French (Canada)

Formatted: French (Canada)

18. Norful AA, Ye S, Van der-Biezen M, Poghosyan L. Nurse Practitioner-Physician Comanagement of Patients in Primary Care. Policy, politics & nursing practice. 2018:1527154418815024.

19. Reich E, Then K, Rankin J. Barriers to Clinical Practice Guideline Implementation for Septic Patients in the Emergency Department. Journal of Emergency Nursing. 2018;44(6):552-62.

20. Kleinpell. Outcome assessment in advanced practice nursing. New York, NY: Springer Publishing Company, LLC; 2017.

21. Smigorowsky MJ, Sebastianski M, Sean McMurtry M, Tsuyuki RT, Norris CM. Outcomes of nurse practitioner-led care in patients with cardiovascular disease: A systematic review and meta-analysis. Journal of advanced nursing. 2020;76(1):81-95.

22. Yang BK, Johantgen ME, Trinkoff AM, Idzik SR, Wince J, Tomlinson C. State Nurse Practitioner Practice Regulations and U.S. Health Care Delivery Outcomes: A Systematic Review. Medical care research and review : MCRR. 2020:1077558719901216.

23. Mares M, McNally S, Fernandez R. Effectiveness of nurse-led cardiac rehabilitation programs following coronary artery bypass graft surgery: a systematic review. 2018;16(12):2304-29.

24. Audet L, Paquette L, Bordeleau S, Lavoie-Tremblay M, Kilpatrick K. The association between advanced practice nursing roles and outcomes in adults following cardiac surgery: A systematic review of randomized controlled trials. International journal of Nursing Studies. 2021.

25. Kwak C, Ko Y. Clinical Nurse Specialist–Led Implementation of an Early Discharge Protocol After Cardiac Surgery. Clin Nurse Spec. 2019;33(4):184-90.

26. Doyle L. A systematic review to enquire is Advanced Nurse Practitioner care more effectivve for the cardiac surgery patient, compared to standard care. European Journal of Cardiovascular Nursing. 2015;1:90.

27. Hall MH, Esposito RA, Jahn L, Blenderman R, Hartman AR, Pekmezaris R, et al. Cardiac surgery nurse practitioner home visits prevent coronary artery bypass graft readmissions. Annals of Thoracic Surgery. 2014;97(5):1488-95.

28. Southey D, Mishra PK, Nevill A, Aktuerk D, Luckraz H. Continuity of care by cardiothoracic nurse practitioners: Impact on outcome. Asian Cardiovascular and Thoracic Annals. 2014;22(8):944-7.

29. Skinner H, Skoyles J, Redfearn S, Jutley R, Mitchell I, Richens D. Advanced care nurse practitioners can safely provide sole resident cover for level three patients: impact

on outcomes, cost and work patterns in a cardiac surgery programme. European Journal Of Cardio-Thoracic Surgery. 2013;43(1):19-22.

30. Bourgon Labelle J, Farand P, Vincelette C, Dumont M, Le Blanc M, Rochefort CM. Validation of an algorithm based on administrative data to detect new onset of atrial fibrillation after cardiac surgery. BMC Medical Research Methodology. 2020;20(1).

31. Needleman J, Buerhaus P, Pankratz VS, Leibson CL, Stevens SR, Harris M. Nurse staffing and inpatient hospital mortality. New England Journal of Medicine. 2011;364(11):1037-45.

32. von Elm E, Altman DG, Egger M, Pocock SJ, Gøtzsche PC, Vandenbroucke JP. The Strengthening the Reporting of Observational Studies in Epidemiology (STROBE) Statement: guidelines for reporting observational studies. International Journal Of Surgery (London, England). 2014;12(12):1495-9.

33. Bourgon Labelle J. Associations entre les pratiques quotidiennes de gestion du personnel de soins et l'incidence de la fibrillation auriculaire postopératoire en chirurgie cardiaque. Savoirs UdeS: Université de Sherbrooke; 2020.

34. Ordre des infirmières et infirmiers du Québec. Encadrement de la pratique des IPS Montréal [internet]. Québec (Can):Ordre des infirmières et infirmiers du Québec;2021 [updated 2021 cited 2021 Aug 24]. Available from: https://www.oiiq.org/en/pratique-professionnelle/pratique-avancee/ips/encadrement-de-la-pratique-des-ips

35. Hoffman LA, Guttendorf J. Integrating Nurse Practitioners into the Critical Care Team. Critical Care Alert. 2014;21(12).

36. Canadian Institute of Health Information. Canadian Coding Standards for ICD-10-CA and CCI [internet].Ottawa (Can):Canadian Institute of Health Information; 2019 [updated 2019 cited 2021 Aug 24]. Available from: https://secure.cihi.ca/estore/productSeries.htm?pc=PCC189.

37. Yenipınar A, Koç Ş, Çanga D, Kaya F. Determining Sample Size in Logistic Regression with G-Power. Black Sea Journal of Engineering and Science. 2019;2(1):16-22.

38. Coleman N, Halas G, Peeler W, Casaclang N, Williamson T, Katz A. From patient care to research: a validation study examining the factors contributing to data quality in a primary care electronic medical record database. BMC Family Practice [Internet]. 2015; 16(1):11.

39. Sudeh C-S, Hardeep S, David R, Jill S, Morris R, Aneez E, et al. Missed diagnostic opportunities and English general practice: a study to determine their incidence, confounding and contributing factors and potential impact on patients through

retrospective review of electronic medical records. Implementation Science [Internet]. 2015; 10(1):105.

40. Tremblay J-A, Laramée P, Lamarche Y, Denault A, Beaubien-Souligny W, Frenette A-J, et al. Potential risks in using midodrine for persistent hypotension after cardiac surgery: a comparative cohort study. Annals of intensive care. 2020;10(1):1-8.

41. Kuhns BD, Hannon CP, Makhni EC, Alter J, Mather RC, 3rd, Salata MJ, et al. A Comparison of Clinical Outcomes After Unilateral or Bilateral Hip Arthroscopic Surgery: Age- and Sex-Matched Cohort Study. The American journal of sports medicine. 2017;45(13):3044-51.

42. Ravi B, Pincus D, Wasserstein D, Govindarajan A, Huang A, Austin PC, et al. Association of Overlapping Surgery With Increased Risk for Complications Following Hip Surgery: A Population-Based, Matched Cohort Study. JAMA internal medicine. 2018;178(1):75-83.

43. Oichi T, Chikuda H, Ohya J, Ohtomo R, Morita K, Matsui H, et al. Mortality and morbidity after spinal surgery in patients with Parkinson's disease: a retrospective matched-pair cohort study. Spine journal. 2017;17(4):531-7.

44. Charlson ME, Pompei P, Ales KL, MacKenzie CR. A new method of classifying prognostic comorbidity in longitudinal studies: development and validation. Journal of chronic diseases. 1987;40(5):373-83.

45. Quan H, Li B, Couris CM, Fushimi K, Graham P, Hider P, et al. Updating and validating the Charlson comorbidity index and score for risk adjustment in hospital discharge abstracts using data from 6 countries. American Journal of Epidemiology. 2011;173(6):676-82.

46. Terwee CB, Prinsen C, Chiarotto A, de Vet H, Bouter LM, Alonso J, et al. COSMIN methodology for assessing the content validity of PROMs–user manual. Amsterdam: VU University Medical Center. 2018.

47. Holt TP, Loraas TM. Using qualtrics panels to source external auditors: A replication study. Journal of Information Systems. 2019;33(1):29-41.

48. Streiner DL, Norman GR, Cairney J. Health measurement scales : a practical guide to their development and use. Oxford: Oxford University Press; 2015.

49. Grant JS, Davis LL. Selection and use of content experts for instrument development. Research in nursing & health. 1997;20(3):269-74.

50. O'Brien SMP, Shahian DMMD, DeLong ERP, Normand S-LTP, Edwards FHMD, Ferraris VAMD, et al. Quality Measurement in Adult Cardiac Surgery: Part 2—Statistical

Formatted: French (Canada)

Formatted: French (Canada)

Considerations in Composite Measure Scoring and Provider Rating. The Annals of Thoracic Surgery. 2007;83(4 Supplement):S13-S26.

51. Shahian D, Edwards F, Ferraris V, Haan C, Rich J, Normand S, et al. Quality Measurement in Adult Cardiac Surgery: Part 1—Conceptual Framework and Measure Selection. The Annals of Thoracic Surgery. 2007;83(4 Supplement):S3-S12.

52. Dubois CA, D'Amour D, Tchouaket E, Clarke S, Rivard M, Blais R. Associations of patient safety outcomes with models of nursing care organization at unit level in hospitals. International journal for quality in health care : journal of the International Society for Quality in Health Care. 2013;25(2):110-7.

53. Gearing RE, Mian IA, Barber J, Ickowicz A. A methodology for conducting retrospective chart review research in child and adolescent psychiatry. Journal of the Canadian Academy of Child and Adolescent Psychiatry. 2006;15(3):126-34.

54. Vassar M, Holzmann M. The retrospective chart review: important methodological considerations. Journal of educational evaluation for health professions. 2013;10:12.

55. McHugh ML. Interrater reliability: the kappa statistic. Biochemia medica. 2012;22(3):276-82.

56. Tchouaket E, Boivin S, Robins S, Sia D, Kilpatrick K, Brousseau S, et al. Development and validation of a time and motion guide to assess the costs of prevention and control interventions for nosocomial infections: A Delphi method among experts. PLoS ONE [Internet]. 2020; 15(11).

57. Rym B, Hendy A, Marine L, Olivier S, Corinne A. Using and reporting the Delphi method for selecting healthcare quality indicators: a systematic review. PLoS ONE [Internet]. 2011; 6(6).

58. Polit DF, Beck CT, Owen SV. Is the CVI an acceptable indicator of content validity? Appraisal and recommendations. Research in Nursing & Health. 2007;30(4):459-67.

59. Vaismoradi M, Salsali M, Turunen H, Bondas T. A qualitative study on Iranian nurses' experiences and perspectives on how to provide safe care in clinical practice. Journal of Research in Nursing. 2013;18(4):351-65.

60. Tabachnick, Fidell. Using multivariate statistics. Sixth edition.. ed. Fidell L, editor: Boston : Pearson Education; 2013.

61. Ibrahim JG, Chu H, Chen M-H. Missing data in clinical studies: issues and methods. J Clin Oncol. 2012;30(26):3297.

62. Armstrong RA. When to use the Bonferroni correction. Ophthalmic & physiological optics : the journal of the British College of Ophthalmic Opticians (Optometrists). 2014;34(5):502-8.

63. Daoud JI, editor Multicollinearity and regression analysis. Journal of Physics: Conference Series; 2017: IOP Publishing.

64. Kilpatrick K, Tchouaket E, Carter N, Bryant-Lukosius D, DiCenso A. Structural and Process Factors That Influence Clinical Nurse Specialist Role Implementation. Clinical Nurse Specialist. 2016;30(2):89-100.

65. Olive DJ. Linear regression. Cham, Switzerland: Springer; 2017.

66. Ramakrishnan N, Hanauer D, Keller B. Mining electronic health records. Computer. 2010;43(10):77-81.
