# Supplementary material for: The level of adherence to best-practice guidelines by interprofessional teams with and without acute care nurse practitioners in cardiac surgery: A study protocol
Source: PLoS One. 2023 Mar 1;18(3):e0282467. doi: 10.1371/journal.pone.0282467 (PMC9976998; doi:10.1371/journal.pone.0282467)
Supplement: Attachment — Submitted filename: Response to Reviewers.docx [file pone.0282467.s007.docx]

**Response to reviewers**

| **Reviewer comment** | **Response** | **Page, line** |
| --- | --- | --- |
| **Reviewer 1** |  |  |
| Please find attached a PDF file with annotations and comments for your consideration. There are some methodological inconsistencies in this manuscript that need to be further clarified. The Data Analysis section is largely repeated in the data collection section and the relevant text for these two sections should be distinct. In part the repetition that is evident may be due to study design considerations. | Thank you for this suggestion. Clarifications and modifications have been made to the manuscript. The data collection and data analysis sections have been modified to avoid repetitions in-between both sections. | p.16 and p. 21-23 |
| I do believe this paper is worthy of publication so ask that you consider the feedback that will strengthen the work and also benefit next steps in terms of conducting the study. | We thank the reviewer for his/her thoughtful revision and comments. | NA |
| In your response I ask that you provide a 3 column table noting the feedback/questions that require a response in the first column, your comments/corrections relevant to that point in the second column and then the page and line number of the change in the next revision. This approach enables a much more efficient review. I look forward to reading your next revision. | Thank you for your revision and comments. The table has been formatted accordingly. | NA |
| Consider starting with 'It is however, unknown whether interprofessional . . .' | Thank you for this recommendation. “*However, it is unknown if interprofessional teams with ACNP are associated with higher levels of adherence to best-practice guidelines compared to interprofessional teams without ACNP*” changed for “*It is however, unknown whether interprofessional teams with ACNP are associated with higher levels of adherence to best-practice guidelines compared to interprofessional teams without ACNP*. “ | p. 2, line 51 |
| By the interprofessional team or the ACNP - it would be useful to specify the perspective from which adherence is being assessed in this sentence for completeness. | Thank you for this suggestion. “*by those teams*” added to “*in order to examine the association between interprofessional teams with and without ACNP, and level of adherence to best-practice guidelines*” | p.3, line 72 |
| Consider revising to 'In 2017 approximately 1 million patients underwent cardiac surgery, the majority of whom reside in developed nations (1). | Thank you for this suggestion. “*In 2017, approximately 1 million patients throughout the world underwent a cardiac surgery (1). Most cardiac surgeries are performed in developed countries (1)*” changed for “*In 2017 approximately one million patients underwent cardiac surgery, the majority of whom reside in developed nations (1)*” | p.4, line 93-94 |
| It would be useful to provide information about the UK/Europe as well given the breadth of the audience that read this journal and those who may be interested in reading your paper. | Thank you for this recommendation. “*In 2014, Germany had the highest rate of coronary revascularization procedures among the European Union with 453 procedures per 100,000 population, followed by Austria (324 procedures per 100,000 population), Croatia (324 procedures per 100,000 population), and Lithuania (319 procedures per 100,000 population (1)*” added to the first paragraph. | p.4, line 110-113 |
| This is the first use of this acronym, so it needs to be spelled out in full here then subsequently abbreviated.  You may wish to confirm formatting requirements with the journal editor but generally if acute care nurse practitioners is being abbreviated to ACNP then there is no need for ACNP's or ACNPs, ACNP should suffice. | Thank you for this suggestion. “*of ACNP*” changed for “*of acute care nurse practitioners (ACNP)*”. The acronym “*ACNP*” was used afterward within the manuscript. | p.6, line 144 |
| the practice or the effect of nurse practitioners? | Thank you for your suggestion. These studies examined the practice of nurse practitioners rather than the effect of NPs. No change was made. | p. 6, line 158 |
| Do you mean expertise in a cognate area of specialty practice? | Thank you for this suggestion. The medical expertise meant the clinical skills and knowledge of the NP practice. Specification and examples have been changed in the manuscript. “*medical expertise*” changed for “*clinical expertise (e.g., prescription of the pharmacotherapy, diagnosis, etc.)*” | p.6, line 168 |
| in rather than to, or do you mean referral to rehab rather than actual participation in rehab? | Thank you for this suggestion. Adjustments have been made to specify the participation of patients in cardiac rehabilitation programs and rather than the referral to cardiac rehabilitation. “*Higher participation of patient to cardiac rehabilitation*” changed for “*higher rates of patients participating in cardiac rehabilitation programs*” | p.7, line 189 |
| reduced | Thank you for this suggestion. “*lower*” changed for “*reduced*” | p.7, line 189 |
| . . . in hospital . . . | “*during their hospital stay*” added to “*patient satisfaction*” | p.7, line 189 |
| In contrast, other . . . | Thank you for this suggestion. “*However*” changed to “*In contrast*”. | p.7, line 191 |
| by | Thank you for this recommendation. “*of Reich et al*.” changed to “*by Reich et al.*” | p.7, line 204 |
| Additionally, to date no validated . . . surgery settings has been located. | Thank you for this suggestion. “*Moreover, until today, no validated extraction tool is available to measure the level of adherence to best-practice guidelines of interprofessional teams with and without ACNP in postoperative cardiac surgery settings*” changed for “*Additionally, to date no validated extraction tool to measure the level of adherence to best-practice guidelines of interprofessional teams with and without ACNP in postoperative cardiac surgery has been located*” | p. 7-8, line 210 |
| challenges | Thank you for this recommendation. “*the difficulty*” changed for “*challenges*” | p.8, line 228 |
| . . . . have used retrospective patient cohorts to develop models adjusted for confounding variables. | Thank you for this suggestion. “*Existing studies (27-30) have used retrospective observational studies to gather a large cohort of patients, limit attrition, and develop statistical models adjusted for many confounding variables.*” changed for “*Existing studies (27-30) have used retrospective patient cohorts to develop models adjusted for confounding variables.* “ | p.8, line 238-239 |
| Given retrospective studies utilise existing data attrition rates cannot be modified so this does not make sense. | Thank you for this suggestion. “*limit attrition*” removed from the sentence. | p.8, line 238-239 |
| . . . mortality risk . . | Thank you for this suggestion. “*risks of mortality*” changed for “*mortality risk*” | p.8, line 240 |
| acute care NP within primary care?? Or do you mean NP within primary care? | Thank you for this suggestion. We refer to ACNP practice within primary care settings outside the hospital setting. Specifications have been added. “*Thirdly, the majority of the RCT focus on ACNP practice within primary care settings (e.g., in-home care, rehabilitation clinic) after the cardiac surgery.*” changed for “*Thirdly, although ACNP usually work within hospital settings, the majority of the RCT focus on ACNP practice within primary care settings (e.g., in-home care, rehabilitation clinic) after the cardiac surgery.*” | p.8, line 242 |
| . . . after surgery, risk reduction and as such the prevention of postoperative complications. | Thank you for this suggestion. “*to ensure the patient's optimal recovery after the surgery, and prevent the risk of adverse events and postoperative complications. Moreover, for patients and families, the acute care hospitalization after the surgery represents a crucial phase of their”* changed for “*to ensure the patient optimal recovery, and prevent the risk of adverse events and postoperative complications. For patients and families, the acute care hospitalization represents a crucial phase of postoperative recovery associated with several physical, psychosocial, and emotional stressors (30)*. “ | p.8-9, line 247-272 |
| The next sentence should be the last in this paragraph as single sentences do not constitute a paragraph. | Thank you for this suggestion. The sentence was merged with the previous paragraph. | p.9, line 275-278 |
| The abstract refers to a single centre but this statement implies that there could be multiple centres involved.  Please specify whether the study is single or multi site in the aim. | Thank you for this suggestion. “*in acute postoperative cardiac surgery settings in Québec, Canada*” changed for “*in an acute postoperative cardiac surgery unit in Québec, Canada*” | p.9, line 277 |
| consider revising this text to read as follows;  It is hypothesised that interprofessional teams with ACNP are associated with . . . | Thank you for this suggestion. “*This study will verify if interprofessional teams with ACNP are associated with a higher level of adherence to best-practice guidelines compared to interprofessional teams without ACNP, after controlling for the patient and interprofessional team characteristics.*” changed for “*It is hypothesized that interprofessional teams with ACNP are associated with a higher level of adherence to best-practice guidelines compared to interprofessional teams without ACNP, after controlling for the patient and interprofessional team characteristics*.” | p.9, line 281-284 |
| . . . within a postoperative . . . | Thank you for this suggestion. “*within postoperative cardiac surgery setting*” changed for “*within a postoperative cardiac surgery setting*” | p.9, line 288 |
| Consider revising as follows as there is repetition in objectives 2 and 3.  2. Compare the characteristics of patients and interprofessional teams according to the presence or absence of ACNP in those teams.  3. Compare level of adherence to best-practice guidelines by interprofessional teams according to the presence or absence of ACNP in those teams. | Thank you for your suggestions. We decided not to make any modification in the study objectives. The objectives have been decided and confirmed with the scientific committee who evaluated the study proposal. The study objectives have also been approved by the ethic research board. | p.9-10 |
| This is best explained in your methods section. | Thank you for your suggestions. We decided not to make any modification in the study hypothesis and objectives. The hypothesis and objectives have been decided and confirmed with the scientific committee who evaluated the study proposal. The study hypothesis and objectives have also been approved by the ethic research board. | p.9-10 |
| The Strengthening . . . Observational Studies (32) will be used as a framework for the conduct of this study (SA appendix). | Thank you for this recommendation. “*This project uses the guidelines from Strengthening the Reporting of Observational Studies in Epidemiology (STROBE) Statement: Guidelines for Reporting Observational Studies (32) were used for this project (SA appendix)*” changed for “*The Strengthening the Reporting of Observational Studies in Epidemiology (STROBE) Statement: Guidelines for Reporting Observational Studies (32) will be used as a framework to conduct this project (SA appendix)*” | p.10, line 315-318 |
| Join with subsequent sentence. | Thank you for this recommendation. “*This study will be conducted at a University healthcare centre (UHC) in Québec, Canada. Annually, this healthcare centre performs approximately 1,000 cardiac surgeries and has a 36-bed postoperative cardiac surgery unit.*” changed to “*This study will be conducted at a University healthcare centre (UHC) in Québec, Canada, who annually performs approximately 1,000 cardiac surgeries and has a 36-bed postoperative cardiac surgery unit.*“ | p.10, line 320-322 |
| retrospective cohort | Thank you for this recommendation. “*dynamic*” changed for “*retrospective*” | p.12, line 372 |
| This information would be best separated.  Please review my next comment regarding the study design. Your approach to sample selection should be explained under the study design sub-heading.  Consider the description of your sample size requirements after you have described the variables of interest identified by your review of the literature as these variables govern sample size requirements for statistical modeling. | Thank you for this suggestion. We decided to keep the sample size calculation in the “Study population and sample section”. The sample size calculation supports the following information regarding the selection and matching of the patients under the care of interprofessional teams with and without ACNP. “*The variables selected for this project are described in the next section*” was added to specify that these variables will be described in the section below. | p.13 |
| This approach impacts on the proposed study design. If you are randomly selecting specific patients and matching them the design is no longer observational.  In my view this is an exploratory descriptive, restrospective cohort study.  Your primary outcome is guideline adherence.  You are grouping patients according to exposure (or not) to an ACNP, and selecting those patients from a specific cohort.  I am not sure of the value in random selection from such a small sample and suspect that it will be very challenging to case match.  In addition, it is not clear what the justification for your sample size calculation is. You note the expected difference in effect size between groups is 15% but have argued that there is no robust evidence to substantiate the difference ACNP make in the cardiac surgery context because there are no existing checklists/benchmarks for this type of assessment. If you wish to retain the 15% difference there needs to be a citation to support the rationale for this difference.  The general rule of thumb in multivariate regression analyses is 20 cases per variable of interest. You have 15 variables of interest so the minimum sample size should be 300 patients.  Within this purposive sample you would be best to identify all patients exposed to the ACNP and then match non-exposed patients with a view to maximising the sample.  I would also consider initially matching according to surgery type as there are known differences in length of stay therefore postoperative recovery between CABGS versus valve patients. If you can match more than 150 patients on the basis of surgery type then attempt to match by sex, then age if you can retain at least 300 patients.  Alternatively you will need to reduce the number of variables in your model. | Thank you for your suggestions. We decided not to make any modification in the study design. The study design has been decided and confirmed with the scientific committee who evaluated the study proposal. A statistician was also implicated in the choice of the study design, the development of the method and the data analyses.  Thank you for this suggestion. The 15% difference was identified with the statistician to encounter a moderate difference, knowing the literature doesn’t have data on the effect of interprofessional teams with and without ACNP on the level of adherence to best-practice guidelines in post-operative cardiac surgery setting. However, a total of 300 patients will be selected, to also encounter the rule of thumb.  Thank you for this recommendation. The three pairing criteria’s (age, sex, type of cardiac procedure) will be used at the same time to match the patients under the care of interprofessional teams with and without ACNP. A pilot study will be conducted to try the matching of the patients with these criteria. The pilot study will inform on the feasibility of the matching of the patients according to the three criteria. Specification has been made within the manuscript to specify that the matching criteria will be used at the same time during the assembly of the retrospective cohort. “*Three criteria (40-43) will be used to match each pair…*” changed for “*Three criteria (40-43) will be used simultaneously to match each pair…*” | p.13, line 404 |
| My understanding of the term practice-guideline is documentation that denotes a specific set of instructions to guide care.  In Figure 1 there are 12 variables in 3 key categories (pharmaco-therapy, lab tests and assessments) and the management of these is considered to be components of best practice or a best-practice recommendation.  I suggest revising the way you refer to these components to reduce the likelihood of confusion for the reader. Recommendations rather than guidelines for best-practice would be optimal. | Thank you for this suggestion. The literature refers to these 12 variables as “best-practice guidelines” (Benjamin et al., 2019; McConnell et al., 2018; STS, 2019). The term “guidelines” has been used widely in the literature versus “recommendation”. Also, within clinical settings, these variables are also known as best-practice guidelines among interprofessional teams and healthcare practitioners. No change was made. | NA |
| . . . and are presented . . . | Thank you for this suggestion. “*identified and presented*” changed for “*identified and are presented*” | p.13, line 414 |
| Is the team the independent variable or the ACNP?  As I understand it, the outcome (adherence measured using the composite score) depends on the ACNP as this is the point of difference in the team. | Thank you for this suggestion. The “interprofessional team with ACNP” is the independent variable. From a clinical point of view, the ACNP collaborates with all members of the interprofessional teams. It is difficult to isolate the practice of ACNP within the interprofessional team because of the interdependence of the ACNP practice with the practice of the whole interprofessional team. This is the reason the independent variable is “*interprofessional teams with ACNP*” rather than “*the practice of ACNP*”. No change was made. | NA |
| How does the inclusion of this measurement link to your aim, objectives and data analysis plan? | Thank you for this recommendation. Measuring the number of days where the ACNP was implicated in the daily follow-up of patients will inform our objective 2, which is the description of the level of adherence to best-practice guidelines by interprofessional teams with and without ACNP. This will allow our team to examine the implication of the ACNP in the patient's hospitalization. For example, was the ACNP doing a daily follow-up of the patient, or was only implicated half of the time during the hospitalization, etc. No change was made within the manuscript. | NA |
| recommendations | Thank you for this suggestion. See answer above. | NA |
| I have concerns about this approach. Generally composite scores are generated on the basis of variable weighting in models rather than score assignment without a theoretical or statistical foundation.  In addition, the scoring does not make sense. A score of 0 is assigned if not medications are provided in the pharmacotherapy variable, but a score of 1 when only 1 of 4 recommended medications is prescribed ignores the other 3 drugs that should be given. Logic indicates that this variable score should range from 0 to 4 with 1 point assigned for each medication. A similar approach would be appropriate for the lab tests and components of assessment.  A statistician should be consulted to provide advice regarding the validity of this approach. | Thank you for your recommendation. The composite score will be calculated for the sum of the scores of each best-practice guideline individually, and not by categories. A single score will be calculated for the 12 best-practice guidelines, based on the achievement of the guidelines (e.g., prescription of the beta blocker) and 2) the associated interventions performed by clinicians (e.g., follow-up of blood pressure). The section describing the development of the composite score was modified accordingly. | p.16, line 479-498 |
| recommendations | Thank you for this suggestion. See answer above. | NA |
| If patients are matched according to surgery type can this still be considered a confounder? A statistician should be consulted to provide advice regarding assessment of matching efficacy and selection of confounders. | Thank you for this suggestion. The type of surgery was selected as a confounding variable to examine if the type of surgery influences the level of adherence to best-practice guidelines by interprofessional teams with and without ACNP. The type of cardiac surgery was also selected as a confounding variable because of its influence on the risk of patient mortality after the surgery (d’Aquino et al., 2017; Lane-Fall et al., 2017). Past studies have shown that patients who underwent a CABG procedure have a higher risk of mortality compared to patients who underwent a cardiac valve replacement or a CABG/valve replacement procedure (d’Aquino et al., 2017). No change was made within the manuscript. | NA |
| Are these secondary outcomes? | Thank you for this suggestion. The length of stay at the hospital and the 30-days readmission at the post-operative cardiac surgery unit are confounding variables rather than secondary outcomes.  The systematic review of Mares et al. (2018) suggested that the prolonged exposition of patients to the ACNP practice is associated with a higher health-related quality of life. In our project, ACNP practice within the post-operative cardiac surgery unit, which will allow our team to examine if a longer or shorter length of stay have an effect on the level of adherence to best-practice guidelines by interprofessional teams with and without ACNP.  Chin et al., 2016 and Sanaiha et al., 2018 support that the 30-days readmission at the post-operative cardiac surgery unit is an important indicator of the quality of care. Within our project, this confounding variable will be collected to examine if patients who were readmitted after their hospital discharge have a lower or higher level of adherence to best-practice guidelines by interprofessional teams with and without ACNP.  No modification was made in the manuscript. | NA |
| There is substantial repetition in the data collection and data analysis section.  Data collection should be focused on extraction and the process of validating the extraction tool separated from the data analysis section that should describe analyses that pertain to testing your hypothesis.  The style of writing in the data analysis section indicates that it has been written independently. | Thank you for this suggestion. The data collection and data analysis sections have been modified to avoid repetitions. | p.16 and p.21-23 |
| If variables in the extraction tool have been identified via a systematic review is this not sufficient justification for the content?  Are the experts being used to assess face validity or criterion validity? | Thank you for this suggestion. The content validity of the extraction tool was conducted with experts to assess the clinical relevance of these items by clinicians practising in postoperative cardiac surgery settings. We believe it was important to assess if these items represented the daily practice of clinicians working bedside with patients who underwent a cardiac surgery. Specifications were made within the manuscript. “*Content validation will be performed following two steps*” changed for “*Content validation will be performed to assess the clinical relevance and the representativeness* *of the practice of interprofessional teams in post-operative cardiac surgery setting of the items of the extraction tool. Content validation will follow two steps (46).*” | p.18, line 547-549 |
| For distribution to whom?  Would it be better to assess inter and intra-rater reliability? | Thank you for this suggestion. The survey will be sent to the experts of the expert committee. Specifications have been added to the manuscript. *“Second, an electronic survey (47) will be created and composed of all items*” changed for “*Second, an electronic survey (47) will be created and sent to each expert of the committee. The survey will be composed…*” | p.18, line 530-531 |
| There are only two steps described. | Thank you for this suggestion. “*three consecutive steps*” changed for “*two consecutive steps*” | p.19, line 541 |
| This seems low | The Cohen Kappa was set according to the COSMIN recommendations (Terwee et al., 2018). A statistician also confirms the choice of the Kappa target value. No change was made within the manuscript. | NA |
| repetitious | Thank you for this suggestion. The additional 30 patients’ health records will be taken from the main retrospective cohort. They are not the same patient’s health records that will be used for the pilot study. Overall, firstly, 30 patients’ health records will be selected and will be used exclusively to conduct the pilot-test. Secondly, another 30 patients’ health records from the main cohort will be used to do a calibration session between the reviewers. Specifications were added to the manuscript for clarification and to avoid repetition.  “*During data collection from the retrospective cohort of patients, an additional 30 patient health records will be reviewed by two independent reviewers to ensure the standardization of the data collection (49, 50). Inter-rater reliability will be assessed and a Cohen Kappa value higher than 0.60 will be targeted (55)*” changed for “*A calibration session among the reviewers will be conducted during the data collection from the main retrospective cohort. A total of 30 patient health records will be reviewed by two independent reviewers (49, 50). Inter-rater reliability will be assessed and a Cohen Kappa value higher than 0.60 will be targeted (55)*” | p.21, line 606-607 |
| Not the presence or absence of the ACNP? | Thank you for this suggestion. “*interprofessional teams-level*” changed for “*interprofessional teams with or without ACNP-level*” | p.21, line 613 |
| This reads as inter-rater reliability assessment to me rather than content validation. | Thank you for this suggestion. The analysis for the content validation was conducted following the COSMIN guidelines and recommendation by a statistician. The following analyses were included as part of the content validation and not the inter-rater reliability because they focus on the clinical relevance of each item assessed by the expert committee. The CVI was calculated to assess if the experts agreed with the inclusion of the items within the extraction tool. The inter-rater Fleiss Kappa was calculated to assess if the experts were in accordance with each other in terms of their assessment. Specifications was added to the “*data collection for content validation*” section to specify the aim of the content validation process. | p.18, line 547-549 |
| How are you differentiating 'gold standard' from best-practice recommendations? What is the gold standard based on? | Thank you for this suggestion. Based on the COSMIN recommendations, the items of the extraction tool were compared with five gold standards. These gold standards are best-practice guidelines published within the literature, supported by robust and high-quality findings. The gold standards of comparison selected for this project were the performance measures of The Society of Thoracic Surgeons. The explanation of the criterion validation process and the selection of the gold standards can be found in page 19-20. Clarifications were added to the data analysis section to specify the gold standards. “*(e.g., the performance measures of the STS)”* added after “*the measured of the gold standard*” | p.22, line 633 |
| Including inferential statistics? | Thank you for this suggestion. The data analysis section has been modified and the inferential statistics section has been moved to Objective 2 “*Bivariate analysis will be conducted, and the statistical significance will be based on a p-value of 0.05 (60). The Bonferroni correction will be applied for the examination of each confounding variable related to the patient and interprofessional teams’ characteristics (62)*.” moved from Objective 3 to Objective 2 section | p.22, line 640-644 |
| How will the daily score be used when calculating the primary outcome? | Thank you for this suggestion. The daily score will be used to calculate the average composite score of the level of adherence to best-practice guidelines by interprofessional teams with and without ACNP. An average of all daily scores during the patient hospitalization will be calculated for each patient of the retrospective cohort. Clarification on the development of the score was made and moved to the data collection section. | p.16, line 479-498 |
| The findings will not be generalizable given the study design. | Thank you for this suggestion. “*and internationally*” removed from “*The findings of this study will further our understanding of the contributions of ACNP in interprofessional teams practising in postoperative cardiac surgery settings in Québec, Canada*” | p.25, line 699 |
| This is an outcome that is already realised, it has been achieved prior to this study so shouldn't be attributed as a potential outcome associated with this work. | Thank you for this suggestion. “*Firstly, as part of this project, a systematic review of randomized controlled trials was published to retrieve the current literature on the efficacy of ACNP and advanced practice nursing roles in postoperative cardiac surgery settings, and to examine their adherence to best-practice guidelines (40).*” removed from the discussion section | p.25, line 701 |
| **Reviewer 2** |  |  |
| The Authors submitted a revised version of their previously submitted Study Protocol paper. The manuscript and the protocollo significantly improved after the revision. I have no further comments or edits. The authors should be commende for their efforts.. | We thank the reviewer for his/her thoughtful revision and comments. | NA |
