# Supplementary material for: The level of adherence to best-practice guidelines by interprofessional teams with and without acute care nurse practitioners in cardiac surgery: A study protocol
Source: PLoS One. 2023 Mar 1;18(3):e0282467. doi: 10.1371/journal.pone.0282467 (PMC9976998; doi:10.1371/journal.pone.0282467)
Supplement: Attachment — Submitted filename: Response to Reviewers.docx [file pone.0282467.s008.docx]

**Response to reviewers**

| **Reviewer comment** | **Response** | **Page, line** |
| --- | --- | --- |
| **Reviewer #2** |  |  |
| The Authors submitted a Study Protocol paper centered on the level of adherence to best-practice guidelines by interprofessional teams with and without acute care nurse practitioners in cardiac surgery. The study would be a retrospective observational study enrolling 300 patients hospitalized between January 1, 2019, and January 31, 2020, in a postoperative cardiac surgery unit in Québec, Canada. To the best of my knowledge, the protocol has its merits. It would represent the first study to examine the level of adherence to best-practice guidelines by interprofessional teams with and without ACNP in a postoperative cardiac surgery setting. | We thank the reviewer for his/her thoughtful revision and comments. | NA |
| The manuscript is well written, even if more attention should be given to English grammar and structure. Some typos should be addressed too (i.e., line 340 double parenthesis after DOAC). | Thank you for this recommendation. The manuscript has been revised and proof-read to correct these errors.  No change was made in line 340. “*DAOC*” is an acronym for “*administration of direct anticoagulant*”, which led us to add a double parenthesis. “*(e.g., administration of direct acting oral anticoagulant (DAOC))”*. | NA |
| The best-practice guidelines the Authors refer to should be explained in the text in extenso and not only in the references. | Thank you for this recommendation. Additional details on each of the three categories of the best-practice guidelines were added to further the description of the best-practice guidelines.  *“The adherence to these four guidelines is defined by the prescription (e.g., beta blocker) and relevant monitoring (e.g., assessment of the blood pressure) of those four medications on a daily basis during the patient's hospitalization and at discharge.”* added next to “*Four best-practice guidelines are included in the category of pharmacotherapy. These guidelines are the prescription and monitoring of medications including: 1) anticoagulants, 2) beta blockers, 3) lipid-lowering agents, and 4) anti-platelets, during hospitalization and at hospital discharge of the patient.*”  “*The monitoring of laboratory tests also includes the interventions conducted by clinicians to ensure the laboratory test values remain within the normal range, such as routinely ordering laboratory tests, assessing laboratory tests to confirm normal values and detect any abnormal values, and performing pharmacological and clinical interventions to address abnormal laboratory test values*.” Added next to “*Four best-practice guidelines on laboratory tests are included, recommending the prescription and monitoring of the following: 1) potassium (K+), 2) magnesium (Mg+), 3) serum glucose, and 4) international normalized ratio (INR) by interprofessional teams during the patient’s hospitalization in the postoperative cardiac surgery unit. The achievement of each best-practice guideline recommendation will be confirmed if the laboratory result falls under the normal range supported by the current literature*.”  “*The postoperative assessments include the assessment of the clinical manifestations, signs, and symptoms experienced by the patient (e.g., patient’s decrease mobility), as well as the interventions conducted by clinicians following these assessments. Interventions include pharmacological interventions (e.g., prescription of analgesic), non-pharmacological interventions (e.g., prescription of a coronary diet), and clinician consultations (e.g., consultation with the nutritionist).* “Added next to “*Four best-practice guidelines are included in the category of the postoperative assessment performed by interprofessional teams. A daily assessment of the patient’s level of pain, surgical wound(s), nutrition, and mobilization will be measured. For each best-practice guideline recommendation, the achievement of the guideline will be confirmed if at least one postoperative assessment per day is performed by the ACNP or other members of the interprofessional teams during the patient’s hospitalization in the postoperative cardiac surgery unit*.” | p. 14, line 342, 345  p.15, line 355-359  p.15-16, line 374-380 |
| Finally, it should be given the measure of the level of adherence or better highlighted since it needs to be clarified. | Thank you for this recommendation. The aim of the project was modified to highlight the measure of the level of adherence to best-practice guidelines. Modifications were made in the abstract and the background sections of the manuscript.  “*This project aims to examine the level of adherence to best-practice guidelines of interprofessional teams with and without ACNP in a postoperative cardiac surgery setting in Québec, Canada*.” changed for “*This project aims to measure and examine the level of adherence to best-practice guidelines of interprofessional teams with and without ACNP in a postoperative cardiac surgery setting in Québec, Canada*.” | p.2, line 55  p.9, line 209 |
| **Reviewer #3** |  |  |
| Greetings!  A well designed protocol. But, the title is too long and confusing.  Best Wishes, | Thank you for your suggestion and revision of our manuscript. We decided not to make any modification in the title. The title has been decided and confirmed with the scientific committee who evaluated the study proposal. The title has also been approved by the ethic research board. | NA |
| **Reviewer #4** |  |  |
| First of all, it was a pleasure reviewing your manuscript. Second, I found the topic to be very interesting. The focus on the roles of ACNPs in medical teams is important and shows the value of these individuals in providing high quality care with better health outcomes without undermining the roles of other healthcare professionals. | Thank you for your revision. | NA |
| Third, I found this protocol to be very detailed and included all necessary information and I am excited to see how this study will turn out. | We thank you for your thoughtful revision. | NA |
| Finally, I only have some comments regarding some the content of the manuscript. In line 137-140, I found it difficult to understand the meaning and purpose of the sentence. I would recommend rewriting this part to make it clear. | Thank you for this suggestion. The sentence was change for clarity purpose. “*NP support care providers in the team with a focus on best evidence is believed to underpin providers’ adherence to best-practice guidelines focused on pharmacotherapy, clinical indicators, and lifestyle promotion*.” changed for “*NP’s evidence-based practice is believed to support care providers’ adherence to best-practice guidelines within the areas of pharmacotherapy, clinical indicators, and lifestyle promotion*.” | p.6, line 137-138 |
| In line 179, there are some typing errors that need adjustment. | Thank you for this suggestion. The sentence was adjusted accordingly. “*Secondly, systematics reviews have highlighted the challenges in adequality controlling confounding variables in existing RCT (21, 23).*” changed for “*Secondly, systematic reviews of randomised controlled trials (RCTs) have revealed that many variables (e.g., severity of illness) influence the effect of ACNP practice on patient and organizational outcomes in postoperative cardiac surgery settings. These variables have not been adequately controlled in current RCTs (21, 23).*” | p.8, line 187-190 |
| In line 525-526, you mentioned the possibility of conducting data transformation in the case of abnormal distribution of the data. I would recommend that you carefully consider this approach as it is a sensitive matter and requires much caution when interpreted. Thank you and please accept my best regards, | Thank you for this comment. Statistical analysis will be conducted in collaboration with a statistician to ensure the rigor and robustness of the manipulation of the data. Interpretation of the findings will be supported with consultations with clinicians to in-depth the understanding of the findings and assess if the findings represent their clinical practice. | NA |
